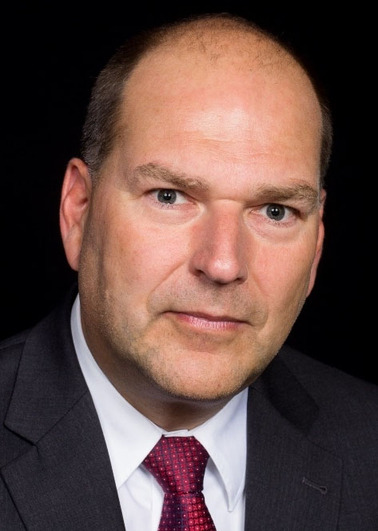# Inorganic Synthesis Based on Reactions of Ionic Liquids and Deep Eutectic Solvents

**DOI:** 10.1002/anie.202104035

**Published:** 2021-07-01

**Authors:** Tao Zhang, Thomas Doert, Hui Wang, Suojiang Zhang, Michael Ruck

**Affiliations:** ^1^ Beijing Key Laboratory of Ionic Liquids Clean Process CAS Key Laboratory of Green Process and Engineering Institute of Process Engineering Chinese Academy of Sciences 100190 Beijing China; ^2^ Innovation Academy for Green Manufacture Chinese Academy of Sciences Beijing 100190 China; ^3^ Faculty of Chemistry and Food Chemistry Technische Universität Dresden 01062 Dresden Germany; ^4^ Max Planck Institute for Chemical Physics of Solids 01187 Dresden Germany

**Keywords:** chemical reactivity, deep eutectic solvents, inorganic materials, ionic liquids, ionothermal synthesis

## Abstract

Ionic liquids and deep eutectic solvents are of growing interest as solvents for the resource‐efficient synthesis of inorganic materials. This Review covers chemical reactions of various deep eutectic solvents and types of ionic liquids, including metal‐containing ionic liquids, [BF_4_]^−^‐ or [PF_6_]^−^‐based ionic liquids, basic ionic liquids, and chalcogen‐containing ionic liquids. Cases in which cations, anions, or both are incorporated into the final products are also included. The purpose of this Review is to raise caution about the chemical reactivity of ionic liquids and deep eutectic solvents and to establish a guide for their proper use.

## Graphical abstract

The chemical reactivity of ionic liquids and deep eutectic solvents can intentionally be exploited as a synthetic tool to prepare inorganic materials that are difficult or even impossible to obtain by traditional synthetic routes. This Review summarizes recent developments in inorganic syntheses that take advantage of reactions with ionic liquids or deep eutectic solvents.

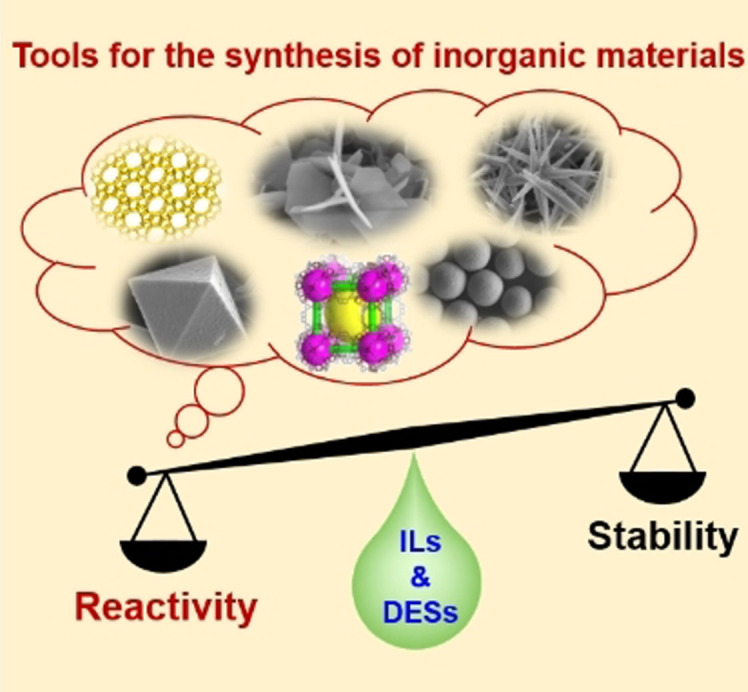

## 1 Introduction

Ionic liquids (ILs), first reported by Paul Walden in 1914,^[1]^ are defined as molten salts with melting points below 100 °C. Nowadays, ILs are widely applied in a broad variety of fields, including catalysis, separations, synthesis, and many others.[^2^, ^3^, ^4^, ^5^] Compared to the broad applications of ILs in organic chemistry, which have already been explored for about 40 years, inorganic syntheses in ILs, especially the so‐called ionothermal syntheses,[^6^, ^7^, ^8^] have been actively investigated only since the early 2000s. Since then, various inorganic compounds (e.g. metals and non‐metals, metal oxides and chalcogenides, metalates and framework compounds) have been prepared using, or in the presence of, ILs.[^9^, ^10^, ^11^, ^12^, ^13^, ^14^]

ILs provide several unique properties for the preparation of inorganic materials. For example, ILs can facilitate the dissolution of versatile precursors, including both inorganic and organic compounds, which is fundamental for the synthesis of most materials.[^15^, ^16^] ILs create a special microphasic separation of the hydrophilic and hydrophobic fragments, typically with imidazolium ILs with their long alkyl chains.^[17]^ This heterogeneity of ILs provides the ability to control nucleation and growth rates, particle sizes, and morphologies in materials synthesis.^[17]^ Furthermore, some other characteristics, such as good thermal stability in ionothermal synthesis, high polarizability for microwave synthesis, or wide electrochemical windows and high conductivity for electrodeposition, make ILs an attractive alternative to conventional organic solvents for the synthesis of inorganic materials as well as to high‐temperature reactions in melts or the solid state.

In 2003, the concept “deep eutectic solvent” was first coined by Abbott et al.^[18]^ Deep eutectic solvents (DESs) are acknowledged as a new class of IL‐analogue solvents and share many characteristics of traditional ILs, such as low vapor pressure, high polarity, and tunable chemical properties. However, DESs are easier to access synthetically. In most cases, a DES is obtained by mixing a quaternary ammonium or phosphonium salt with a hydrogen‐bond donor (HBD), thereby generating a new liquid phase with a melting point below that of either individual component.[^19^, ^20^] No purification is usually needed. Furthermore, most DESs are quite inexpensive because of the low cost of their constituents, such as urea and choline chloride. Therefore, IL analogues as well as the more accessible DESs are increasingly being used in the synthesis of inorganic materials.

The thermal and chemical stabilities of ILs are usually highlighted as advantageous for inorganic synthesis. Previous investigations have indicated that the actual degradation temperature of ILs is overestimated by the onset decomposition temperature (*T*_onset_) derived from the ramped temperature in thermogravimetric analysis.[^21^, ^22^, ^23^] Therefore, the concept of long‐term thermal stability is utilized to obtain more accurate information on the decomposition of ILs at high temperature. The thermal stability of ILs, including the characterization methods, mechanism of decomposition, and kinetics of thermal degradation, has been extensively explored by several research groups,[^24^, ^25^, ^26^, ^27^, ^28^, ^29^] and is not within the scope of this Review.

ILs are readily accessible as inert reaction media for inorganic synthesis. Dai and co‐workers demonstrated that several imidazolium ILs containing [NTf_2_]^−^ anions could be used as the flux medium for the direct recycling of spent cathode materials or as effective structure‐directing templates for the synthesis of advanced catalysts and anode materials because of their good thermal and chemical stability.[^30^, ^31^, ^32^] In all cases, the [NTf_2_]^−^‐containing imidazolium ILs can be readily reused and recycled after the reaction, thus providing new strategies for designing sustainable ILs for advanced inorganic synthesis.

However, many ILs contain reactive moieties in either the cation or anion. Thus, the ILs themselves can take part in reactions. For example, an IL can be tailored for a specific task, such as to release one component of the desired product upon its decomposition. Thus, the IL acts as solvent, template, and reactant, thereby simplifying the reaction system significantly. In other circumstances, the IL cation and anion can separate during the reactions, thereby leading to incorporation of the IL cation or anion in the final products. Such reactions are often used in the synthesis of some framework compounds (e.g. zeolites, MOFs, and polycationic/polyanionic compounds).[^13^, ^33^, ^34^] The IL cation or anion serves as a counterion to balance the charge of the framework as well as a template. IL decomposition or cation/anion separation during the ionothermal synthesis may cause a change in the properties of the IL (e.g. viscosity, conductivity, and dissolving capacity) and further influence the formation of the target product.^[35]^ Thus, the reaction mechanism of ILs should be considered. Furthermore, the ionothermal approach, in particular, which exploits the chemical reactivity of ILs or DESs, provides new options for the synthesis of inorganic materials.

Several inspiring reviews on this young and fast‐growing subject of inorganic synthesis in ILs or DESs have been published, with an emphasis on selected themes.[^7^, ^8^, ^9^, ^10^, ^11^, ^36^, ^37^, ^38^, ^39^, ^40^] However, most of them lack a comprehensive understanding of the chemical reactivity of the ILs or DESs in the reactions. To date, there are only a few case studies on the detailed reaction mechanisms.[^16^, ^41^, ^42^, ^43^, ^44^, ^45^] In comparison, the chemical reactivity of ILs in organic synthesis has been discussed in several reviews.[^35^, ^46^, ^47^, ^48^]

Herein, we attempt to systematically and comprehensively summarize this fascinating research area from the point of view of inorganic synthesis based on the chemical reactions of ILs or DESs. It includes reactions of metal‐containing ILs, fluorine‐containing ILs, basic ILs, chalcogen‐containing ILs, and DESs. Moreover, reactions of ILs whose cations, anions, or both are incorporated into the final products are also included. Table 1 shows a summary of all the abbreviations used in this Review. The decomposition and reaction mechanism of some IL/DES‐based reactions are discussed. This Review aims to illustrate a promising synthetic approach based on the reactivity of ILs/DESs and to provide a better understanding of the fundamental chemistry of ILs/DESs in the reactions.

**Table 1:** The abbreviations of the IL cations and anions as well as other reagents used in this Review.

Abbreviation	Full name
[MIm]^+^	1‐methylimidazolium cation
[MMIm]^+^	1,3‐dimethylimidazolium cation
[EMIm]^+^	1‐ethyl‐3‐methylimidazolium cation
[BMIm]^+^	1‐butyl‐3‐methylimidazolium cation
[PMIm]^+^	1‐pentyl‐3‐methylimidazolium cation
[OMIm]^+^	1‐methyl‐3‐octylimidazolium cation
[C_16_MIm]^+^	1‐hexadecyl‐3‐methylimidazolium cation
[BMMIm]^+^	1‐butyl‐2,3‐dimethylimidazolium cation
[C_12_MMIm]^+^	1‐dodecyl‐2,3‐dimethylimidazolium cation
[BMPyr]^+^	1‐butyl‐1‐methylpyrrolidinium cation
[DMPyr]^+^	1‐decyl‐1‐methylpyrrolidinium cation
[P_4444_]^+^	tetrabutylphosphonium cation
[P_66614_]^+^	trihexyltetradecylphosphonium cation
[NTf_2_]^−^	bis(trifluoromethylsulfonyl)imide anion
[OTf]^−^	triflate anion
TBAH	tetrabutylammonium hydroxide
TEAH	tetraethylammonium hydroxide
BTMAH	benzyltrimethylammonium hydroxide
TBPH	tetrabutylphosphonium hydroxide
Pbis	1,5‐bis(3‐methylimidazole‐2‐selone)pentane
Me	methyl group
Et	ethyl group
*t*‐Bu	*tert*‐butyl group
DBU	1,8‐diazabicyclo(5.4.0)undec‐7‐ene

## 2 Reactions of Metal‐Containing Ionic Liquids

An overview of all the discussed studies reporting the synthesis of inorganic materials using, or in the presence of, ILs is presented in Table 2.

**Table 2:** A summary of all the studies on inorganic materials prepared from ionic liquids discussed in this Review.

Material composition	Ionic liquid	Refs.
CuCl	[C_5_H_5_N‐C_12_H_25_][CuCl_4_]	[50]
ZnO	Zn(L)_4_(NTf_2_)_2_ (L=alkylamine)	[51]
CuO	[C_16_MIm]_2_[CuCl_4_]	[52]
CuS	[BMIm]_2_[Cu_2_Cl_6_]	[53]
ZnS	[C_*n*_MIm][ZnCl_3_] (*n*=4, 8, and 16)	[54]
FeS_2_	[C_12_MMIm][ FeCl_4_]	[55]
A_2_SiF_6_ (A=Li, Na, K, Rb, and Cs)	[BMIm][PF_6_]	[68]
MnF_2_	[BMIm][BF_4_]	[69]
iron fluoride	[BMIm][BF_4_]	[70, 71, 72, 73]
NaYF_4_	[BMIm][BF_4_], [BMIm][PF_6_]	[74]
Ce^3+^‐, Tb^3+^‐, Eu^3+^/Sm^3+^‐doped BaLuF_5_	[OMIm][PF_6_]	[75]
Eu^2+^‐doped BaFCl	[BMIm][BF_4_]	[76]
Yb^3+^‐, Er^3+^/Tm^3+^‐doped NaYF_4_	[BMIm][BF_4_]	[77]
MF_*x*_, M=Fe, Co, Pr, Eu, Gd, and Er	[BMIm][BF_4_]	[78, 79, 80]
turbostratic boron nitride (t‐BN)	[BMMIm][BF_4_]	[81]
ZnO	TBAH, TEAH, and BTMAH	[83, 84, 85, 86], [89, 90]
metal (hydr)oxides, metal=Fe, Co, Mn, Ni, Cu	TBAH	[87, 88]
SrTiO_3_	TBAH, TBPH	[91]
CdSe, Bi_2_Se_3_, ZnSe, and PbSe	Pbis	[109]
NiSe_2_, ZnSe, Bi_2_Te_3_, Ag_2_Te, and Te nanostructures	[P_66614_]Cl, [P_66614_][decanoate], and [P_66614_][N(CN)_2_]	[110, 111, 112]
CdS	[BMIm][SCN]	[113]
ZnSe, Cu_2−*x*_Se, and CdSe	[BMIm][SeO_2_(OCH_3_)]	[115, 116, 117]
ZnSe	selenoether‐based ILs^[a]^	[118]
BiOCl	[C_16_MIm]Cl	[122]
Li_4_B_7_O_12_X (X=Cl, Br)	[P_66614_]X (X=Cl, Br)	[123]
A_2_B_5_O_9_X (A=Sr, Ba, X=Cl, Br)	mixture of [P_66614_]X and LiNTf_2_ (X=Cl, Br)	[124]
Pb_2_B_5_O_9_X (X=Cl, Br)	[P_66614_]X without adding LiNTf_2_ (X=Cl, Br)	[125]
SIZ‐1, SIZ‐3, SIZ‐4, and SIZ‐5	[EMIm]Br	[6]
metal phosphates (metal=Be, Al, Zn, and Fe)	[MIm][H_2_PO_4_]	[134, 135]
[Si_48_O_96_]F_4_(C_8_N_2_H_15_)_2_(C_2_H_7_O)_2_	[BMIm]OH_0.65_Br_0.35_	[136]
porous TiNb_2_O_7_ and MnCeO_x_	[BMIm][NTf_2_]	[30, 31]
[EMIm][Cd(btc)]^[b]^	[EMIm]Br	[138]
[Cd_3_F(ina)_4_(4‐pic)_3_][BF_4_], [Cd_3_F(ina)_3_(4,4′‐bpy)_2_(4‐pic)_2_][BF_4_]_2_⋅(4,4′‐bpy)⋅2 H_2_O, and [Cd_3_F(ina)_3_(4,4′‐bpy)_3_][BF_4_]_2_⋅(4,4′‐bpy)⋅2 H_2_O^[c]^	[BMIm][BF_4_]	[149]
[PMIm][Zn_2_(btc)(OH)Br]	[PMIm]Br	[151]
[BMIm][Zn_2_(btc)(OH)I]	[BMIm]I	[152]
[BMPyr]_2_[Br_20_]	mixture of [DMPyr]Br and [BMPyr][OTf]	[158]
[P_4444_]_2_[Br_24_]	mixture of [P_4444_]Br and [P_66614_][NTf_2_]	[159]
[BMMIm]_24_[Sn_36_Ge_24_Se_132_] and [BMIm]_24_[Sn_32.5_Ge_27.5_Se_132_]	([BMMIm][BF_4_] and [BMIm][BF_4_]	[164]
[Sb_10_Se_10_][AlCl_4_]_2_	[BMIm]Cl⋅*n* AlCl_3_	[166]
[Sb_2_Se_2_][AlCl_4_] and [Sb_13_Se_16_][AlCl_4_]_6_Al_2_Cl_7_	[BMIm]Cl⋅4.7 AlCl_3_	[167, 168]
[Sb_13_Se_16_Br_2_][AlX_4_]_5_	[BMIm]Br⋅5.1 AlCl_3_	[169]
[Sb_7_Se_8_Br_2_][Sb_13_Se_16_Br_2_][AlBr_4_]_8_	[BMIm]Br⋅4.7 AlBr_3_	[169]
[Sb_7_Se_8_Br_2_][AlX_4_]_3_	[BMIm]Br⋅4.7 AlBr_3_ (a small amount of NbCl_5_)	[169]
(CuBi_8_)[AlCl_4_]_2_[Al_2_Cl_7_] and (CuBi_8_)[AlCl_4_]_3_	[BMIm]Cl⋅4 AlCl_3_	[170]
Ni_2_P and Ni_12_P_5_	[P_4444_]Cl	[188]
Co_2_P	[P_66614_]_2_[CoCl_4_]	[189, 190]

[a] ILs of *N*‐[(phenylseleno)methylene]pyridinium, *N*‐(methyl)‐ and *N*‐(butyl)‐*N*′‐[(phenylseleno)methylene]imidazolium with [NTf_2_]^−^ anions. [b] btc=benzene‐1,3,5‐tricarboxylate. [c] ina=isonicotinate, 4,4′‐bpy=4,4′‐bipyridine, 4‐pic=4‐methylpyridine.

In this Review, metal‐containing ionic liquids (M‐ILs) represent a subclass of ILs that contain a metal atom as part of the cation and/or anion. In addition to the general fluidic properties of ILs, the incorporated metal ions endow M‐ILs with some new functions, such as luminescent, catalytic, or magnetic properties. Recently, M‐ILs have gained increasing research attention in a variety of fields (e.g. catalysis, optical devices, and magnetic components).^[49]^

**Figure 1:**
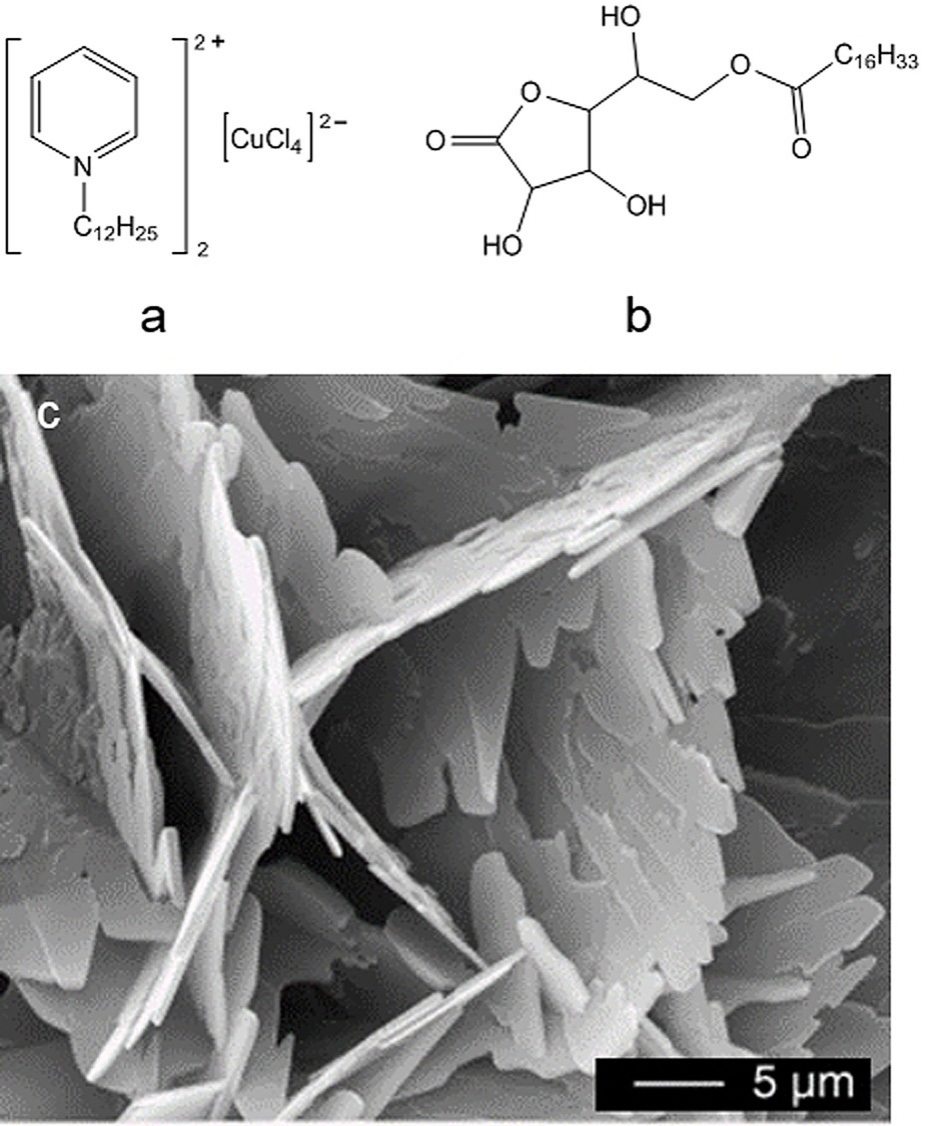
a) A Cu‐containing IL. b) 6‐*O*‐palmitoyl ascorbic acid. c) SEM image of CuCl nanoplatelets precipitated from a 1:1 (w/w) mixture of (a) and (b) at 85 °C. Reproduced with permission.^[50]^ Copyright 2004, Wiley‐VCH.

M‐ILs that serve as metal sources in inorganic synthesis have been widely investigated. In 2004, Taubert reported that CuCl nanoplatelets were synthesized from a Cu‐containing IL (Figure 1 a) and 6‐*O*‐palmitoyl ascorbic acid (Figure 1 b).^[50]^ It was found that the mixture of the two compounds forms thermotropic liquid crystals with lamellar self‐assembled structures. The layered structures then template the formation of CuCl nanoplatelets as the temperature is increased (Figure 1 c). The Cu‐containing IL can be regarded as an “all‐in‐one” IL because it acts as solvent, reactant, as well as template. After this study, a number of M‐ILs were designed and applied for the synthesis of inorganic nanomaterials, including metal oxides and metal sulfides.

Dai and co‐workers reported that hierarchical ZnO structures with diverse morphologies were obtained under an ionothermal synthesis when employing the Zn‐containing IL Zn(L)_4_(NTf_2_)_2_ (L=alkylamine, NTf_2_=^−^N(SO_2_CF_3_)_2_) as both the solvent and Zn source.^[51]^ The solvent properties can be tailored by varying the IL ligand structures, thereby resulting in various morphologies of ZnO. In addition, CuO nanorods can be prepared in the presence of the Cu‐IL [C_16_MIm]_2_[CuCl_4_] under solvothermal conditions.^[52]^

Zheng and co‐workers demonstrated that three‐dimensional (3D) hierarchical CuS microspheres assembled from nanosheets were produced from the Cu‐containing IL precursor [BMIm]_2_[Cu_2_Cl_6_] by a solvothermal method.^[53]^ The Cu‐IL plays a significant role in directing the final CuS structures. On one hand, the crystal growth along the [001] direction is inhibited because the [BMIm]^+^ prefers to adsorb onto the (001) facets of CuS. On the other hand, the assembly of CuS microspheres is influenced by the alkyl chain of the Cu‐IL. A tight hierarchical CuS structure is the preferred form when a short‐chain IL is used. ZnS quantum dots were prepared using the Zn‐containing IL [C_*n*_MIm][ZnCl_3_] (*n*=4, 8, and 16) as a precursor, template, and solvent.^[54]^ FeS_2_ microspheres wrapped by N‐doped reduced graphene oxide were synthesized from the Fe‐based IL [C_12_MMIm][ FeCl_4_].^[55]^ The Fe‐IL can be used as the metal and nitrogen source, an assembly medium, and surfactant.

Another major application of M‐ILs is for the low‐temperature electrodeposition of various metals and alloys.[^56^, ^57^, ^58^, ^59^] One well‐studied example involves the electrodeposition of Al in the IL‐AlCl_3_ system, whose Lewis acidity depends on the molar ratio of the organic salts to metal halides.^[56]^ In recent years, ILs with metal‐containing cations have been developed for the electrodeposition of metals at high current densities because of the easy access of cationic metal complexes to the electrode surface.^[60]^ Investigations into the electrodeposition of metals (e.g. Ni, Co, Cu, Al, and rare earth metals) by various cationic metal‐containing ILs have been reported by a series of research groups.[^60^, ^61^, ^62^, ^63^, ^64^, ^65^]

## 3 Reactions of [BF_4_]^−^‐ or [PF_6_]^−^‐Based Ionic Liquids

Inorganic metal fluorides are well‐studied for their applications in photonics, catalysis, biosensing, lubricants, electrochemical energy storage, and high‐temperature superconductor devices.[^66^, ^67^] In traditional syntheses of metal fluorides, the toxic and harmful HF, NaF, or NH_4_F is usually utilized as a fluorine source. Recent studies have shown that fluorine‐containing ([BF_4_]^−^ or [PF_6_]^−^) ILs can be used as fluorine sources, thereby opening a safe pathway to prepare metal fluorides with novel morphologies and functions. The hydrolysis of the [BF_4_]^−^ anion occurs in the presence of a small amount of residual water in the IL or the water of crystallization in metal salts upon heating, thereby forming BF_3_⋅H_2_O and F^−^. The reaction of fluoride ions with metal ions under the given conditions contributes to the in situ crystallization of metal fluorides.^[67]^ Similarly, [PF_6_]^−^ may also decompose to release F^−^ under specific conditions.^[68]^

**Figure 2:**
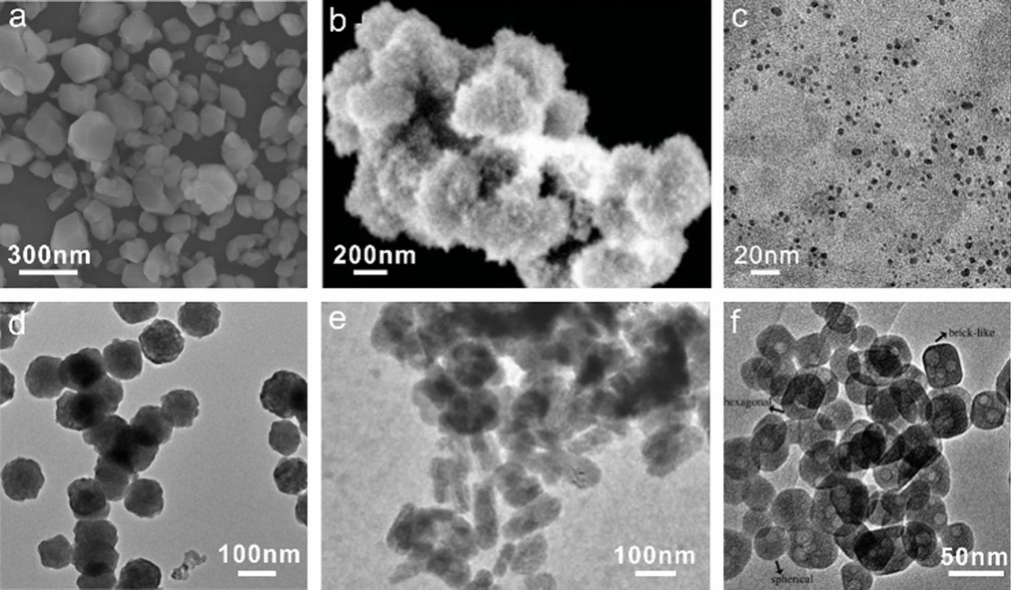
SEM or TEM images of metal fluorides obtained in (or in the presence of) [BF_4_]^−^‐ or [PF_6_]^−^‐based ILs. a) The as‐prepared MnF_2_ sample. Reproduced with permission.^[69]^ Copyright 2015, Wiley‐VCH. b) An orthorhombic FeF_3_⋅0.33H_2_O electrode fabricated in [BMIm][BF_4_]. Reproduced with permission.^[70]^ Copyright 2010, Wiley‐VCH. c) Na_2_SiF_6_ nanoparticles obtained from a mixture of [BMIm][PF_6_] and ethanol under microwave irradiation. Reproduced with permission.^[68]^ Copyright 2017, Wiley‐VCH. d) A BaLuF_5_ sample synthesized in a mixture of ethylene glycol and [OMIm][PF_6_]. Reproduced with permission.^[75]^ Copyright 2017, Elsevier Inc. e) BaFCl:Eu^2+^ nanoparticles. Reproduced with permission.^[76]^ Copyright 2018, Wiley‐VCH. f) NaY_0.78_Yb_0.20_Er_0.02_F_4_ nanocrystals. Reproduced with permission.^[77]^ Copyright 2009, Royal Society of Chemistry.

Wen and co‐workers synthesized a nanostructured MnF_2_ by using Mn(CH_3_COO)_2_⋅4 H_2_O as a manganese source and [BMIm][BF_4_] as a fluorine source (Figure 2 a).^[69]^ The resulting MnF_2_ nanoparticles could be promising anode materials for lithium batteries with a long cycle life. Li et al. reported that a variety of hydrated Fe‐based fluoride nanoparticles could be successfully synthesized using Fe(NO_3_)_3_⋅9 H_2_O and [BMIm][BF_4_] as the precursors (e.g. Figure 2 b).[^70^, ^71^, ^72^, ^73^] In the process, [BMIm][BF_4_] serves as the solvent, template, and fluorine source. The as‐synthesized iron‐based fluoride and its composite materials can be used as cathodes for lithium or sodium batteries.

Wickleder and co‐workers synthesized a series of ternary fluoridosilicates A_2_SiF_6_ (A=Li, Na, K, Rb, and Cs) with particle sizes of a few tens of nanometers (Figure 2 c) by using [BMIm][PF_6_] as both the solvent and fluoride source in a microwave‐assisted ionothermal synthesis at low temperatures.^[68]^ This approach is very simple, energy‐efficient, and time‐saving since it avoids the use of harmful and toxic HF or its derivatives. The nanoparticles of this series of ternary fluoridosilicates A_2_SiF_6_ are regarded as possible host materials for future LEDs.

Rare‐earth fluorides are an interesting family of compounds because of their optical properties, and their syntheses in ILs have been widely investigated. Yan and co‐workers reported that novel spherical NaYF_4_ nanoclusters were prepared in [BMIm][BF_4_] or [BMIm][PF_6_] with the assistance of microwave radiation.^[74]^ ILs serve as reaction solvents, fluorine sources, and microwave absorbents. They found that some NaF particles were formed when [BMIm][PF_6_] was used to prepare NaYF_4_ nanocrystals. This result is probably due to the easier breaking of the P−F bond in [BMIm][PF_6_] compared to the B−F bond in [BMIm][BF_4_]. Thus, a high concentration of fluoride ions in the [BMIm][PF_6_] reaction system leads to the formation of NaF. The morphology of the NaYF_4_ nanoparticles obtained in [BMIm][PF_6_] is different from that obtained in [BMIm][BF_4_]. The main reason is that the higher viscosity of [BMIm][PF_6_] might prevent the assembly and aggregation of small nanoparticles, which affects the final morphology. Finally, NaYF_4_ nanoparticles doped with lanthanide ions (Ln^3+^) display superior upconversion properties.

Zou and co‐workers reported that BaLuF_5_:Ce,Tb,Eu(Sm) sub‐microspheres with tunable sizes and morphologies could be synthesized from IL/ethylene glycol dual‐solvent systems (Figure 2 d).^[75]^ The results show that the [OMIm][PF_6_] IL used serves as both the fluoride source and capping agent. The aggregation manner of the nanoparticles and the size of the particles can be tuned by changing the ratio of the IL and ethylene glycol. Doping with Ce^3+^, Tb^3+^, or Eu^3+^/Sm^3+^ ions allows the photoluminescence colors to be tuned from green, through yellow, to orange. Wickleder and co‐workers reported a novel IL‐assisted synthesis of Eu^2+^‐doped BaFCl nanoparticles by using [BMIm][BF_4_] as both the solvent and fluorine source under sonochemical and microwave conditions (Figure 2 e).^[76]^ Additionally, the [BMIm][BF_4_] IL coordinates the Eu^2+^ ions and stabilizes their oxidation state. Kong and co‐workers reported the ionothermal synthesis of pure‐phase NaYF_4_:Yb^3+^,Er^3+^/Tm^3+^ nanoparticles (Figure 2 f).^[77]^ The key in the synthesis is the use of [BMIm][BF_4_], which serves as the solvent, template, and fluorine source. The obtained nanocrystals can be directly dispersed in water and present—because of the hydrophilic overlayer of [BMIm][BF_4_] on the crystal surface—a strong positive charge as well as strong upconversion luminescence.

Janiak and co‐workers reported that a variety of transition‐metal and rare‐earth metal fluoride nanoparticles (MF_*x*_‐NPs, M=Fe, Co, Pr, Eu, Gd, and Er) could be obtained by the microwave‐induced decomposition of their metal amidinate complexes [M{MeC(N(*i*Pr)_2_)}_*n*_] (M(amd)_*n*_; M=Mn^II^, Fe^II^, Co^II^, Pr^III^, Gd^III^, Er^III^) and tris(2,2,6,6‐tetramethyl‐3,5‐heptanedionato)europium(III) (Eu(dpm)_3_) in [BMIm][BF_4_].[^78^, ^79^, ^80^] The M(amd)_*n*_ and Eu(dpm)_3_ are suspended in dried [BMIm][BF_4_] under argon. An increased temperature drives the decomposition of M(amd)_*n*_/Eu(dpm)_3_ to release the metal species and the hydrolysis/decomposition of the [BF_4_]^−^ anion to release F^−^. The reaction of metal species with F^−^ results in the formation of metal fluorides.

Interestingly, it is found that the degraded components of [BF_4_]^−^ anions can be utilized in the presence of moisture or heat as a boron precursor in the reaction. Turbostratic boron nitride nanoflakes (t‐BN) were obtained on using [BMMIm][BF_4_] as the boron source.^[81]^ The hydrogen‐bond‐co‐π‐π stack mechanism is responsible for the self‐assembly of the [BMMIm][BF_4_] IL for the formation of the flake‐like t‐BN.

## 4 Reactions of Basic Ionic Liquids

Conventional inorganic bases, such as NaOH, KOH, or Na_2_CO_3_, have many disadvantages such as being corrosive and producing waste. Basic ILs, which combine the advantages of inorganic bases and ILs, have great potential to replace them. They are noncorrosive, nonvolatile, flexible, and immiscible with many organic solvents. Therefore, basic ILs are often applied in some base‐catalyzed processes in organic chemistry^[82]^ and nanomaterial preparation. In inorganic synthesis, basic IL anions provide the required basic environment of the reactions, while the organic cations may play an important role in the crystal nucleation and growth.

**Figure 3:**
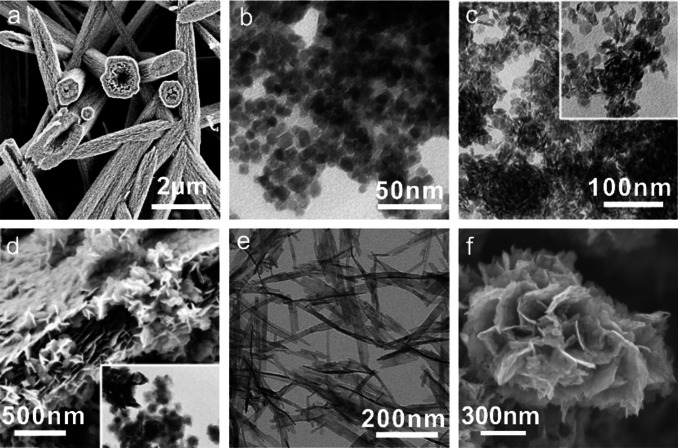
SEM and TEM images of metal oxide and hydroxide samples obtained in (or in the presence of) TBAH. a) Hollow ZnO mesocrystals. Reproduced with permission.^[86]^ Copyright 2008, Wiley‐VCH. b–d) γ‐Fe_2_O_3_/Fe_3_O_4_ cubes and spheres, β‐Ni(OH)_2_ plates, and Co(OH)_2_ plates, respectively. Reproduced with permission.^[87]^ Copyright 2008, Wiley‐VCH. e) CuO nanoplates. Reproduced with permission.^[88]^ Copyright 2008, American Chemical Society. f) Hierarchically structured SrTiO_3_ microparticles. Reproduced with permission.^[91]^ Copyright 2017, Royal Society of Chemistry.

Li et al. designed a variety of tetraalkylammonium hydroxide ILs to synthesize metal oxides of various sizes and shapes. For example, several unusual ZnO nanostructures, including flower‐like particles, “lotus‐leaf‐like” ZnO plates, and porous ZnO plates, can be produced from tetrabutylammonium hydroxide (TBAH).^[83]^ In these reactions, TBAH serves as an efficient IL precursor for the synthesis of ZnO nanoparticles with controlled sizes and morphologies.[^84^, ^85^] Interestingly, not only regular nanocrystals, but also special hollow ZnO mesocrystals can be obtained in TBAH by varying the concentration of the zinc acetate precursor (Figure 3 a).^[86]^ The resulting tubular microstructures are composed of smaller nanosized ZnO primary particles with a high degree of order, which classifies them as mesocrystals. It is assumed that the large tetrabutylammonium cation of TBAH reverses the polarity of the negatively charged surfaces of the small particles, thus preventing further growth of these small primary particles and aiding their aggregation into larger structures. A wide variety of uniform metal (hydr)oxide particles (Figure 3 b–e) was successfully prepared from water/TBAH liquid precursor mixtures simply by using metal acetates (M(OAc)_2_, M=Fe, Co, Mn, Ni, and Cu) as the metal sources.[^87^, ^88^] Moreover, some other types of hydroxide‐based IL precursors, including tetraethylammonium hydroxide (TEAH) and benzyltrimethylammonium hydroxide (BTMAH), were used instead of TBAH for the efficient synthesis of ZnO particles with controlled sizes and morphologies.[^89^, ^90^]

Ruck and co‐workers successfully extended the use of TBAH for the fabrication of a perovskite‐type oxide SrTiO_3_ (Figure 3 f).^[91]^ Hierarchical desert‐rose‐like SrTiO_3_ microstructures with a high surface area of up to 186 m^2^ g^−1^ are obtained by using TBAH as the alkali under solvothermal conditions mediated by ethylene glycol. It was found that TBAH or tetrabutylphosphonium hydroxide (TBPH) can replace the ethylene glycol and act as both the solvent and reactant to yield polyhedral SrTiO_3_ nanoparticles.

## 5 Reactions of Chalcogen‐Containing Ionic Liquids (Including Reactions of Ionic Liquids with Chalcogens)

This section covers reactions of imidazolium or phosphonium salts with chalcogens (S, Se, or Te) to generate the corresponding imidazole‐2‐chalcogenones or trialkylphosphane chalcogenides, respectively. The reaction mechanisms are also discussed. The utilization of chalcogenones or trialkylphosphane chalcogenides as chalcogen sources for the synthesis of metal chalcogenides is also summarized in this section.

**Scheme 1:**
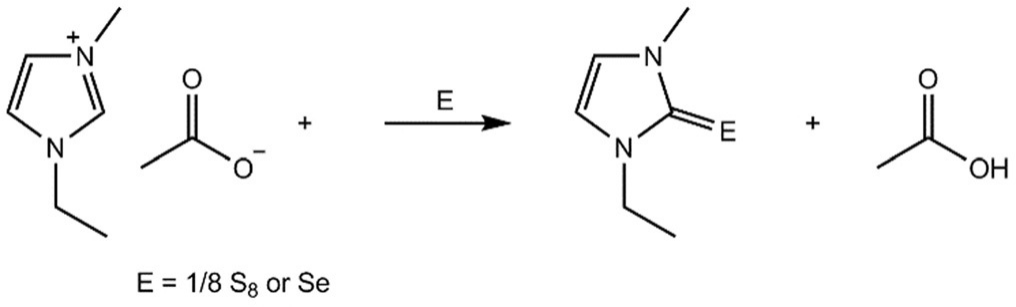
Reactions of the imidazolium acetate ionic liquid with chalcogens.^[93]^.

It has been recognized that the C2‐position of the 1,3‐dialkylimidazolium cation contains an acidic proton. Deprotonation of this position leads to the formation of a stable carbene, which is often used as an intermediate in organic synthesis.^[92]^ It is found that reactions of inorganic reactants (e.g. chalcogens) with imidazolium salts at their C2‐position can also take place, especially in the presence of a base. Rogers and co‐workers reported that 1,3‐dialkylimidazolium acetates can react with elemental S or Se to afford the corresponding imidazole‐2‐chalcogenones directly, even in the absence of an additional base (Scheme 1).^[93]^ Investigations show that the imidazolium acetate IL serves as both the carbene source and base to generate, in situ, the carbenes, which then react with S to form the thione.

However, when other anion‐based (e.g. Cl^−^, [HSO_4_]^−^, [SCN]^−^, [NTf_2_]^−^, and [BF_4_]^−^) imidazolium ILs were tested, no reactions with the chalcogens were observed. In these cases, ultrasound irradiation or strong bases are usually required. Ansell et al. demonstrated that [MMIm]I can readily react with elemental S to yield 1,3‐dimethylimidazole‐2‐thione in the presence of K_2_CO_3_ in methanol.^[94]^ This “MeOH/K_2_CO_3_” method has since been utilized to prepare a variety of chalcogenones, including 1,3‐dialkylimidazole‐2‐thione/selone,[^95^, ^96^] bridged bis(imidazoline‐2‐thione/selone),[^97^, ^98^, ^99^] and bridged mixed bidentate N‐heterocyclic carbene (NHC)/sulfur ligands,^[100]^ starting from the corresponding imidazolium salts and chalcogens. Other “base/solvent” systems such as MeOH/pyridine/DBU,^[101]^ pyridine/Et_3_N,^[102]^ and THF/KO(*t*‐Bu)^[103]^ have also been investigated for the synthesis of imidazole‐2‐chalcogenones by the reaction of quaternary imidazolium salts with chalcogens.

The use of refluxing methods has also been reported. For example, Wasserscheid and co‐workers found that 1,3‐dialkylimidazolium halide salts can react with elemental S in refluxing methanol in the presence of NaOMe to afford 1‐alkyl‐3‐methylimidazolium‐2‐thiones.^[104]^ Tian et al. prepared a variety of selenones by refluxing the respective imidazolium salts with Se and Na_2_CO_3_ in water.^[105]^

In addition, Inesi and co‐workers developed an efficient combined electrochemical and ultrasound method for the synthesis of imidazole‐2‐thiones.^[106]^ In this reaction, the imidazolium IL is first electrochemically reduced to the corresponding carbene, which then reacts with elemental S under ultrasound irradiation to give the target thiones in high yields. Lei and co‐workers reported that reactions of imidazolium salts with potassium thioacetate/thiocyanate as the S source yield imidazole‐2‐thiones rapidly and efficiently under microwave radiation.^[107]^

In contrast to thiones and selenones, the synthesis of tellurones derived from their corresponding imidazolium salts is much more difficult because of the relatively weak C−Te bond compared to C−S/Se bond. Singh and co‐workers developed a new approach for the high‐yielding synthesis of benzimidazolin‐2‐tellurones by the reaction of Te nucleophiles Na_2_Te/Na_2_Te_2_ with various benzimidazolium salts.^[108]^ Compared to Te powder, the stronger Te^2−^/Te_2_^2−^ nucleophiles facilitate chemical reactions with benzimidazolium salts under mild conditions.

The use of imidazole‐2‐chalcogenones as chalcogen precursors for the synthesis of metal chalcogenides is rare.^[109]^ Shi and co‐workers utilized 1,5‐bis(3‐methylimidazole‐2‐selone)pentane (Pbis) as a novel Se precursor to successfully synthesize a series of metal selenides, including ZnSe, CdSe, PbSe, and Bi_2_Se_3_.^[109]^ Pbis is easily obtained and air‐stable. Moreover, the significantly positively charged 1,5‐bis(3‐methylimidazole)pentane and the negative Se valence in Pbis lead to the rapid and efficient reaction of Pbis with metal cations. Thus, it constitutes a facile and general method to prepare various metal selenide nanoparticles.

**Figure 4:**
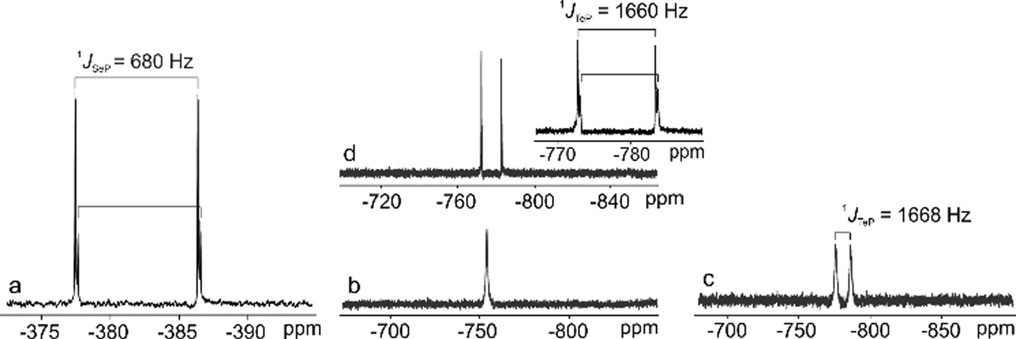
a) ^77^Se NMR spectrum of the reaction solution with a molar ratio of Se/[P_66614_][decanoate]=1:4 at 220 °C under Ar. Reproduced with permission.^[110]^ Copyright 2017, Royal Society of Chemistry. ^125^Te NMR spectra of Te solutions with a molar ratio of Te/[P_66614_][decanoate]=1:7.6 (b), 1:2 (c), and 1:1 (d) at 220 °C under Ar. Reproduced with permission.^[111]^ Copyright 2018, Wiley‐VCH.

In comparison to the popularly investigated imidazolium ILs, phosphonium ILs have been less studied. Despite their higher thermal and chemical stability, phosphonium ILs are not completely inert, and decomposition can occur under certain conditions.^[35]^ Ruck and co‐workers showed that quaternary phosphonium cations of ILs can undergo decomposition in the presence of Se/Te above 220 °C.[^110^, ^111^] A series of dissolution tests, in which the solute Se/Te species were tracked by nuclear magnetic resonance (NMR) spectroscopy, was applied to systematically investigate the decomposition mechanisms. These studies indicate that one alkyl substituent of the quaternary phosphonium cations is eliminated through an S_N_2 decomposition pathway, leading to dissolution of Se/Te through the formation of the corresponding trialkylphosphane selenides/tellurides (Figure 4). However, the decomposition mechanism of the phosphonium IL in the presence of Te is much more complicated than that in the presence of Se. The ^1^ *J*_PTe_ coupling, which indicates a P−Te bond is formed, is only observed in the NMR spectra when a sufficient amount of Te (e.g. Te/IL=1:1) is present (Figure 4 d). The use of smaller amounts of Te results in the ^125^Te satellites in the ^31^P NMR spectra disappearing and the doublets in the ^125^Te NMR spectra collapsing to one broad doublet (Figure 4 c) or a single line (Figure 4 b). In addition, the existence of a parallel, competitive IL decomposition route to the S_N_2 reaction is regarded as the side reaction for the dissolution of Te. This may at least partially explain the relatively lower solubility of Te compared to Se in phosphonium‐based ILs.

**Figure 5:**
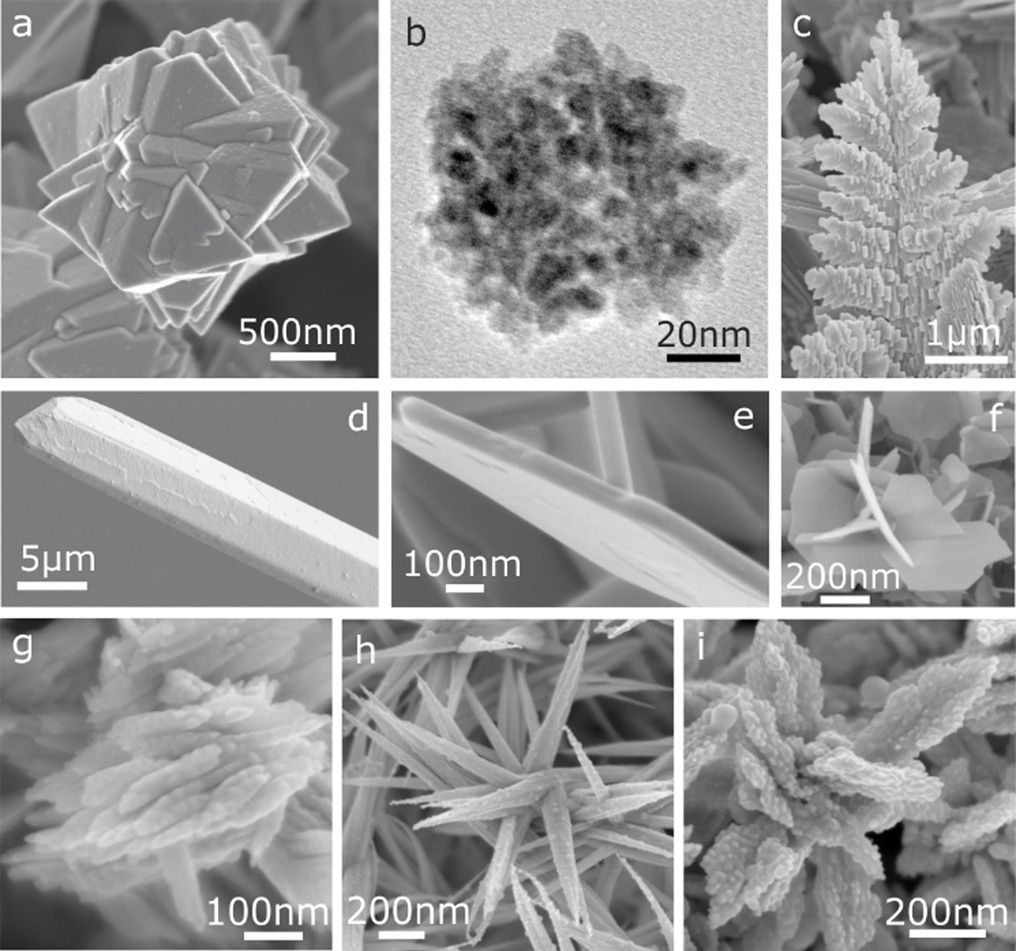
SEM and TEM images of Se/Te‐based nano‐/microparticles obtained in phosphonium ILs. a,b) NiSe_2_ and ZnSe nanoparticle aggregates. Reproduced with permission.^[110]^ Copyright 2017, Royal Society of Chemistry. c) Leaf‐like Te microstructure. d) Te single crystal. e) Bi_2_Te_3_ nanoplate. f) Flower‐like Bi_2_Te_3_ particle. Reproduced with permission.^[111]^ Copyright 2018, Wiley‐VCH. g–i) 3D complex Te microstructures. Reproduced with permission.^[100]^ Copyright 2020, Royal Society of Chemistry.

These preformed trialkylphosphane selenides/tellurides can serve as Se/Te reservoirs for the preparation of nanostructured metal selenides/tellurides, such as octahedral NiSe_2_ particles, ZnSe nanocrystal aggregates, or 3D intergrown Bi_2_Te_3_ crystals (Figure 5 a,b,e,f). Additionally, Te single crystals and various Te microstructures, including 3D hierarchical fern‐leaf‐like Te structures, 3D Te fusiform assemblies, and 3D aloe‐like Te microarchitectures, are obtained when using a reactive Te solution in dried commercial [P_66614_]Cl as the Te precursor (Figure 5 c,d,g–i).[^111^, ^112^] These IL‐based synthetic methods provide convenient and efficient strategies for the preparation of Se/Te‐based materials compared to the conventional complicated solution methods which typically need a reductant (e.g. NaBH_4_) in the presence of a surfactant (e.g. polyvinylpyrrolidone or cetyltrimethylammonium bromide).

Some examples also document the direct synthesis of chalcogenides from chalcogen‐containing ILs. This case is similar to the use of metal‐containing ILs as metal sources, as mentioned above, since both the chalcogen and metal species can be incorporated in the IL anion or cation. Wu and co‐workers reported that the thiocyanate IL [BMIm][SCN] can serve as both the solvent and sulfur source for the preparation of CdS nanocomposites.^[113]^ Zheng and co‐workers designed the Se‐containing IL [BMIm][SeO_2_(OCH_3_)] as a novel Se source.^[114]^ The IL anion ([SeO_2_(OCH_3_)]^−^ ion) shows similar reactivity as the commonly used Na_2_SeO_3_ system. However, precipitates form in some systems from the reaction of Na_2_SeO_3_ with metal ions. The use of the [SeO_2_(OCH_3_)]^−^ anion avoids this precipitation problem, because of its weaker polarizing capability, and the metal ions exist as free ions in the solutions. Moreover, particle growth is influenced by the adsorption of the [BMIm]^+^ cation on the formed crystal surfaces, leading to nanoparticles with diverse shapes. As a consequence of these distinct characteristics of this Se‐containing IL precursor, various metal selenides with special morphologies, including CuSe nanoflakes,^[115]^ Cu_2−*x*_Se nanocrystals,^[115]^ ZnSe hollow nanospheres,^[116]^ and CdSe nanospheres and nanodendrites,^[117]^ have been successfully prepared. Selenium can also be incorporated into the cation of the ILs. Janiak and co‐workers synthesized several selenoether‐functionalized ILs with the [NTf_2_]^−^ anion, and these were used as both the reaction media and Se reagents for the preparation of ZnSe nanoparticles under irradiation with microwaves at 220 or 250 °C.^[118]^ It is assumed that the proximity of the in situ generated carbene precursor complex to the Zn^2+^ ions leads to an interaction and the formation of an intramolecular coordinative Zn−Se bond. The decomposition of these NHC(Se)‐Zn complexes under microwave heating yields the corresponding ZnSe nanoparticles. Furthermore, the same group reported the synthesis of a variety of metal selenide (e.g. CdSe, PbSe, and Pd_17_Se_15_) nanoparticles by decomposing the corresponding metal‐Se‐based molecular complexes in ILs.[^119^, ^120^, ^121^]

## 6 Reactions of Ionic Liquids Whose Cations, Anions, or Both Are Incorporated into the Final Products

ILs allow the creation of several types of open‐framework materials such as zeolites or metal‐organic frameworks (MOFs) as well as polyanionic/polycationic compounds. In many reactions, the IL cation or anion, as the counterion, is incorporated into the final structures as a result of the charge of the framework. In this section, reactions of ILs whose cations, anions, or both are incorporated into the final products are summarized.

### 6.1 Metal Halide Compounds

As new types of halide sources, halide‐based ILs exhibit distinctive features compared to the commonly used halide sources. Zheng and co‐workers reported that various BiOCl nanostructures, such as ultrathin BiOCl nanoflakes, curved nanoplates, and nanoplate arrays, could be successfully prepared using [C_16_MIm]Cl as the solvent, template, and chloride source.^[122]^ The [C_16_MIm]^+^ cation with its long alkyl chain tends to adsorb on the (001) plane of BiOCl, and crystal growth along the *c*‐axis direction is inhibited, which leads to the formation of thin BiOCl nanoflakes. The obtained BiOCl nanoplates show potential applications for the removal of heavy metal ions in the field of wastewater treatment.

**Figure 6:**
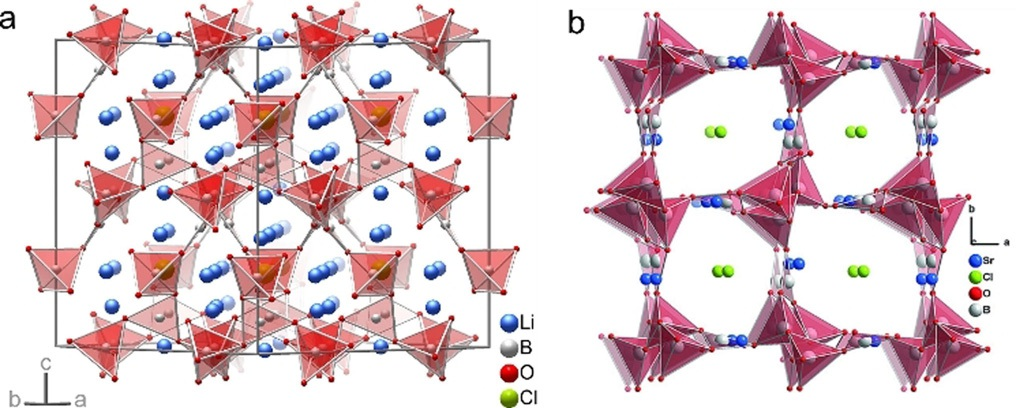
a) The crystal structure of Li_4_B_7_O_12_Cl. Reproduced with permission.^[123]^ Copyright 2019, American Chemical Society. b) The crystal structure of Sr_2_B_5_O_9_Cl. Reproduced with permission.^[124]^ Copyright 2020, Wiley‐VCH. The [BO_4_] tetrahedra and [BO_3_] triangles in the borate framework are highlighted in red.

Ruck and co‐workers investigated the ionothermal synthesis of several borate halide compounds using quaternary phosphonium halide ILs as both the solvent and halide source. The Li‐ion‐conductive polycrystalline Li_4_B_7_O_12_Cl can be obtained in [P_66614_]Cl in the presence of lithium acetate dehydrate and boric acid at a temperature of 200 °C for 48 h.^[123]^ The borate framework, constructed from [BO_4_] tetrahedra and [BO_3_] triangles, forms interpenetrating channels, within which Li^+^ and Cl^−^ are trapped (Figure 6 a). Similarly, Li_4_B_7_O_12_Br can also be synthesized using [P_66614_]Br as the bromide source. Three nanostructured borate halides of the A_2_B_5_O_9_X type (A=Sr, Ba, X=Cl, Br)—Sr_2_B_5_O_9_Cl nanorods, Sr_2_B_5_O_9_Br nanoneedles, and Ba_2_B_5_O_9_Cl nanosheets—were synthesized by the reaction of acetates A(OAc)_2_ and boric acid B(OH)_3_ in a mixture of [P_66614_]X and LiNTf_2_.^[124]^ As shown in Figure 6 b, the [BO_4_] tetrahedra and [BO_3_] triangles form the anionic [B_5_O_9_]^3−^ framework with large channels. Halide anions, from the phosphonium halide ILs, and metal cations are trapped in these channels. Investigations further show that the reactivity of [P_66614_]X is promoted by adding the metal salt LiNTf_2_ to the reaction system, as it weakens the cation–anion interactions of the ILs. However, microcrystalline Pb_2_B_5_O_9_X (X=Cl, Br), with an average diameter of 1 μm, was obtained in [P_66614_]X without adding LiNTf_2_.^[125]^ Both Pb^2+^ and X^−^ are incorporated into the channels of Pb_2_B_5_O_9_X compounds. These borate halides show efficient second harmonic generation, even as microcrystalline powders.

### 6.2 Zeolites

Zeolites are a family of porous materials that are widely applied in adsorption and catalysis.^[126]^ As an example, the industrially significant aluminosilicate zeolite frameworks are comprised of corner‐sharing [SiO_4/2_] and [AlO_4/2_]^−^ tetrahedra linked through bridging oxygen atoms. Thus, the overall framework bears a negative charge caused by the negatively charged [AlO_4/2_]^−^ units. A wide range of cations, such as Na^+^, K^+^, Ca^2+^, Mg^2+^, and others, can be accommodated in the zeolite cavities as counterions to balance the anionic charge. If a zeolite is prepared in an IL, the organic cations are incorporated into the zeolite channels to balance the charge and also act as structural templates.^[34]^

**Figure 7:**
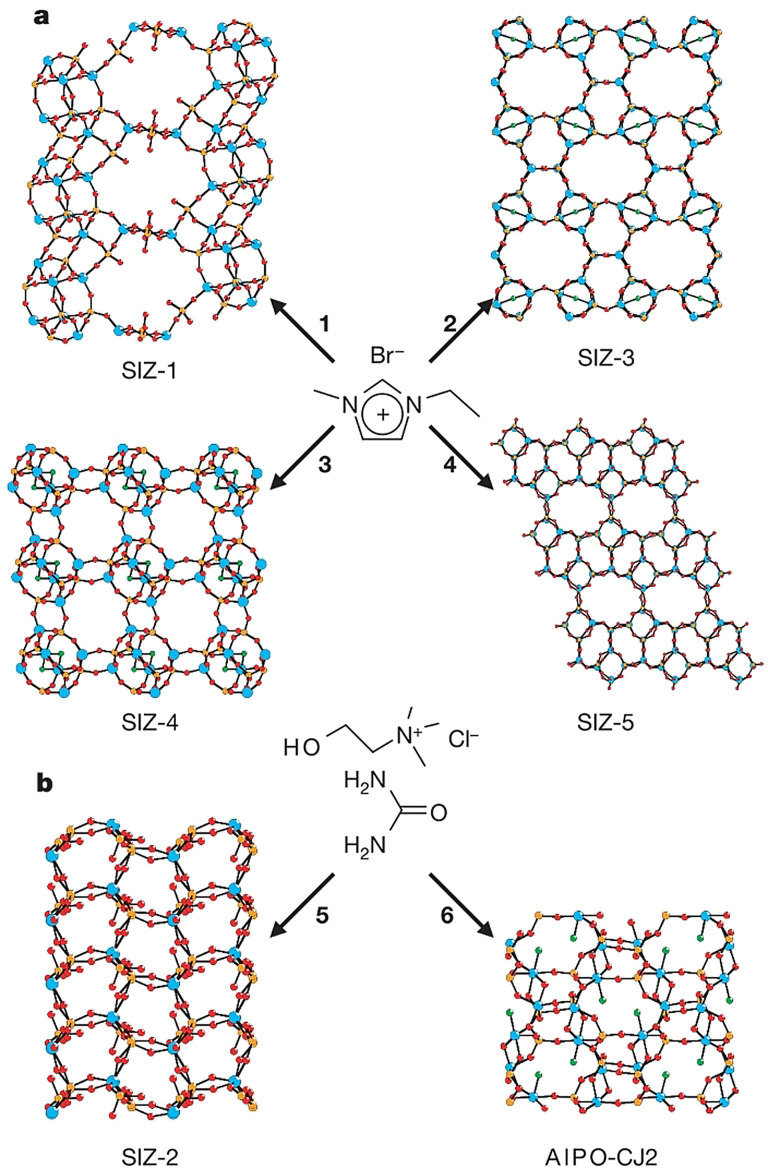
Aluminophosphate materials synthesized in ILs and DESs. a) SIZ‐1, SIZ‐3, SIZ‐4, and SIZ‐5 are obtained ionothermally in [EMIm]Br. b) SIZ‐2 and AlPO‐CJ2 synthesized in a ChCl/urea mixture.^[6]^ Reproduced with permission from the Nature Publishing Group. Copyright 2004.

The first IL‐based synthesis of zeolites in ILs was reported by Morris and co‐workers in 2004.^[6]^ Several aluminophosphates (e.g. SIZ‐1, SIZ‐3, SIZ‐4, and SIZ‐5, Figure 7 a) are produced in [EMIm]Br. In SIZ‐1 (Figure 7 a), the hexagonal prismatic units are joined to form layers, and the neighboring layers are linked by four tetrahedral centers into a 3D framework. The framework is negatively charged due to the presence of terminal P−O bonds. [EMIm]^+^ cations are present in the pores, balancing the negative framework charges as well as templating the formation of the zeolite structure.

Since then, various framework types have been successfully obtained for aluminophosphates, including AEL,[^127^, ^128^, ^129^] LTA,[^130^, ^131^] CHA,^[132]^ and layered structures,^[133]^ by the ionothermal synthetic route. In these reactions, the organic cations of the IL have been demonstrated to be effective templates that often reside in the cavities of the obtained zeolites to compensate for the negative charges of the frameworks. Very recently, Lin and co‐workers designed the multifunctional IL [MIm][H_2_PO_4_] (MIm=*N*‐methylimidazolium) for the ionothermal synthesis of crystalline metal phosphates (metal=Be, Al, Zn, and Fe).[^134^, ^135^] Interestingly, [MIm][H_2_PO_4_] provides the phosphorus source to build the framework unit without phosphoric acid, as well as being a template and solvent. The obtained aluminum phosphate has a 2D structure with 8‐membered‐ring windows and the beryllium phosphate contains extra‐large 24‐membered‐ring channels. The [MIm]^+^ cations are located within the large channels in the case of beryllium phosphate or within the interlayer region in the case of aluminum phosphate.

Despite much success in the ionothermal synthesis of aluminophosphates, the application of ILs in the preparation of silica‐based zeolites still faces a major obstacle because silica is poorly soluble in ILs. Morris and co‐workers designed a task‐specific IL [BMIm]OH_0.65_Br_0.35_ for the first ionothermal synthesis of siliceous zeolites.^[136]^ The hydroxide component in the IL anion leads to a better dissolution of silica, while the cation templates the formation of the MFI framework. Fluoride is added to promote the dissolution of silicate precursors and the crystallization of the zeolite. The fluorine atoms are present in the final product as part of SiO_4_F within the pentasil units. The [BMIm]^+^ cations are incorporated inside the pores to balance the negative charge of the fluoride.

The IL‐templating effects were also used by Dai and co‐workers to synthesize porous transition‐metal oxides.[^30^, ^31^] Unlike the incorporation of the IL cation or anion into the zeolite framework, the IL template (e.g. [BMIm][NTf_2_]) used for the fabrication of porous transition‐metal oxides can be easily extracted and removed by organic solvents. Based on this [BMIm][NTf_2_] templating method, well‐defined nanoporous TiNb_2_O_7_ and mesoporous MnCeO_*x*_ have been synthesized that exhibit superior performance for fast‐rechargeable lithium‐ion batteries and high activity for the selective oxidation of hydrocarbons at low temperature (100–120 °C), respectively.

### 6.3 Metal‐Organic Frameworks (MOFs)

MOFs, as a group of porous crystalline materials consisting of metal ions (clusters) coordinated to organic ligands, have received much research interest in many applications (e.g. gas storage, gas separation, catalysis, drug delivery, and sensing) because of their diverse structures, high porosity, and controllable chemical structures.^[137]^ In an ionothermal synthesis of MOFs, the IL cation, anion, or both may be incorporated in the open cavities of MOFs.

When anionic MOF frameworks are created in ILs, it is common for the IL cation to be incorporated into the MOF structure to balance the negative charge of the framework and to also act as a template.^[34]^ For example, the metal‐organic framework [EMIm][Cd(btc)] (btc=benzene‐1,3,5‐tricarboxylate) was obtained by the reaction of Cd(NO_3_)_2_⋅4 H_2_O with H_3_btc using [EMIm]Br as a reaction medium. The framework is composed of Cd_2_ units with six btc ligands coordinated to each Cd_2_ moiety, while each btc is linked by three Cd_2_ units. This leads to the formation of an anionic [Cd(btc)]^−^ framework. The [EMIm]^+^ cations are located in the void space of the framework.^[138]^ If H_3_btc is reacted with other metal precursors (e.g. Mn(OAc)_2_⋅4 H_2_O, Ni(OAc)_2_⋅4 H_2_O, or Co(OAc)_2_⋅4 H_2_O) in [C_*n*_MIm]X (*n*=2 or 3; X=Cl^−^, Br^−^, or I^−^) the corresponding anionic frameworks ([Mn(btc)]^−^, [Ni_3_(btc)_2_(OAc)_2_]^2−^, or [Co_3_(btc)_2_(OAc)_2_]^2−^) are obtained, with 1‐alkyl‐3‐methylimidazolium cations within the channels.[^139^, ^140^] A series of MOFs containing other linking groups, such as bdc (benzene‐1,4‐dicarboxylate),[^141^, ^142^] iso‐bdc (benzene‐1,3‐dicarboxylate),[^143^, ^144^] 1,4‐ndc (naphthalene‐1,4‐dicarboxylate),^[145]^ btetc (benzene‐1,2,4,5‐tetracarboxylate),^[146]^ and d‐cam (d‐camphorate)[^147^, ^148^] has been successfully prepared in various imidazolium ILs, with the cations residing in the open regions of the frameworks.

The anions of an IL can also be incorporated in the voids of the MOF as charge‐compensating species. Huang and co‐workers reported that three Cd_3_F‐based compounds with cationic frameworks, namely [Cd_3_F(ina)_4_(4‐pic)_3_][BF_4_], [Cd_3_F(ina)_3_(4,4′‐bpy)_2_(4‐pic)_2_][BF_4_]_2_⋅(4,4′‐bpy)⋅2 H_2_O, and [Cd_3_F(ina)_3_(4,4′‐bpy)_3_][BF_4_]_2_⋅(4,4′‐bpy)⋅2 H_2_O (ina=isonicotinate, 4,4′‐bpy=4,4′‐bipyridine, 4‐pic=4‐methylpyridine) were prepared in [BMIm][BF_4_].^[149]^ The [BF_4_]^−^ anion serves as a charge‐balancing unit located in the voids of the frameworks. However, the F^−^ ions formed by in situ hydrolysis of the [BF_4_]^−^ anion are trapped within the MOF framework through the formation of a [Cd_3_F]^5+^ unit. Thus, the [BMIm][BF_4_] IL serves as solvent, fluoride source, and structure‐directing agent.

In another case, the anion of an IL could be utilized as the reactant through coordinating with metal ions to form a negatively charged framework, while the cation was trapped in the channels as a counterion and a template, that is, both the anion and cation of the IL can be incorporated in the voids of MOF frameworks during ionothermal syntheses.^[150]^ Kwon and co‐workers reported the synthesis of [PMIm][Zn_2_(btc)(OH)Br] in [PMIm]Br and [BMIm][Zn_2_(btc)(OH)I] in [BMIm]I by using Zn(NO_3_)_2_⋅6 H_2_O and H_3_btc as starting materials.[^151^, ^152^] In both cases, the IL anions (bromide or iodide) form Zn−X (X=Br or I) bonds to become part of the anionic frameworks. The imidazolium cations are incorporated in the channels and appear to show strong interactions with the frameworks.

### 6.4 Polyanionic/Polycationic Compounds

Polyanions and polycations constitute an interesting class of compounds as a consequence of their diverse structures and chemical bonding. The ionothermal approach is promising for the formation of new polyanionic and polycationic compounds with unique structures that are not accessible using well‐established hydro‐/solvothermal techniques. Recently, the application of room‐temperature ILs for the preparation of polycationic or polyanionic cluster compounds was extensively explored by the groups of Ruck, Dehnen, Kanatzidis, Feldmann, and Riedel.[^13^, ^36^, ^153^, ^154^, ^155^, ^156^, ^157^]

**Figure 8:**
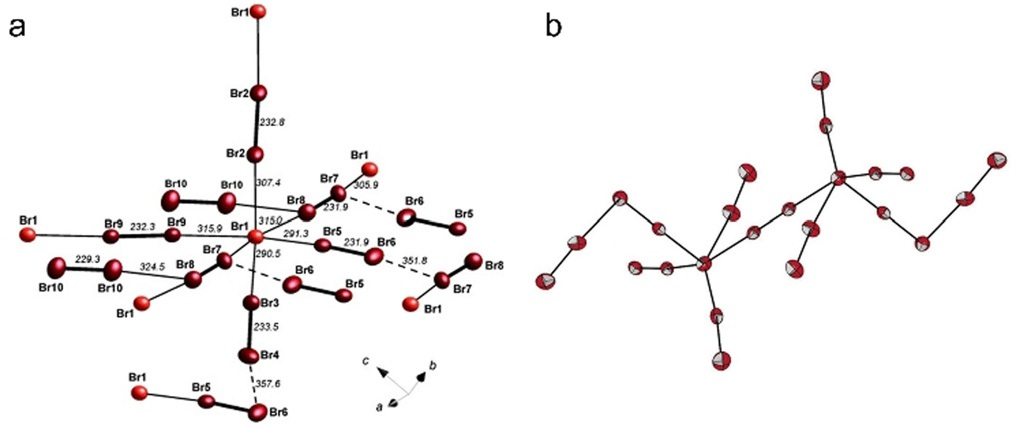
a) Structure of [Br_20_]^2−^ in [C_4_Mpyr]_2_[Br_20_]. Cations are omitted for clarity. Reproduced with permission.^[158]^ Copyright 2011 Wiley‐VCH. b) Structure of the highest known polybromide anion [Br_24_]^2−^ in [P_4444_]_2_[Br_24_]. Cations are omitted for clarity. Reproduced with permission.[^159^, ^160^] Copyright 2015 and 2020 Wiley‐VCH.

To date, several types of polyanionic compounds, such as polyhalides,[^158^, ^159^, ^160^] metal carbonyl cluster anions,[^161^, ^162^, ^163^] and anionic chalcogenide frameworks,[^36^, ^164^, ^165^] have been synthesized in ILs. For example, polybromides were usually limited to a maximum of 10 atoms because of their increasing vapor pressure and reactivity before the new IL‐based approaches were developed.^[13]^ The use of ILs has resulted in several new polybromides. Feldmann and co‐workers reported the first 3D bromine‐rich polybromide network, namely [BMPyr]_2_[Br_20_], through an ionothermal synthesis using a mixture of [DMPyr]Br and [BMPyr][OTf] (Figure 8 a).^[158]^ [DMPyr]Br acts as a “bromide donor” to bromine molecules. [BMPyr][OTf] is used as a “liquifier” to form a eutectic mixture with [DMPyr]Br, thereby establishing a liquid state of the mixture at or even below room temperature for convenient isolation of the solid product. Later, Maschmeyer and co‐workers discovered that the higher‐order polybromide [P_4444_]_2_[Br_24_] can be obtained from an equimolar mixture of [P_4444_]Br and [P_66614_][NTf_2_] (Figure 8 b).^[159]^ Investigations show that the IL cation is the dominant factor in the product‐selective synthesis. The large number of close H⋅⋅⋅Br interactions between the butyl chains of [P_4444_]^+^ and the [Br_24_]^2−^ are assumed to stabilize and direct the formation of this high‐nuclearity species. In [P_4444_]_2_[Br_24_], the “central” bromine atom is five‐coordinate, whereas that of [BMPyr]_2_[Br_20_] is six‐coordinate (Figure 8). This arises primarily from the required space for an octahedral building block being occupied by a butyl chain of [P_4444_]^+^, which prevents coordination of a sixth dibromine molecule.

In another case reported by Dehnen and co‐workers, two polyanionic compounds [BMMIm]_24_[Sn_36_Ge_24_Se_132_] and [BMIm]_24_[Sn_32.5_Ge_27.5_Se_132_] were synthesized by the reaction of [K_4_(H_2_O)_3_][Ge_4_Se_10_] with SnCl_4_⋅5 H_2_O in tetrafluoridoborate ILs ([BMMIm][BF_4_] and [BMIm][BF_4_], respectively) in the presence of DMMP (DMMP=2,6‐dimethylmorpholine).^[164]^ These two compounds contain the largest known discrete polyanion [Sn_36−*x*_Ge_24+*x*_Se_132_]^24−^ (*x=*0 or 3.5) with an outer diameter of 2.83 nm and an inner diameter of 1.16 nm. The IL cations [BMMIm]^+^ and [BMIm]^+^ serve as counterions that surround the anions and partially penetrate them. This research further shows that the addition of a small amount of an amine promotes phase formation and phase selectivity of the products.

Polycationic compounds are commonly carried out in Lewis‐acidic ILs, which usually combine alkylimidazolium halides with more than equimolar amounts of aluminum or gallium trihalides (MX_3_).^[13]^ This leads to the high solubility of metals (e.g. Se, Te, Sb, and In) and their metal halides in highly polar Lewis‐acidic systems. Moreover, the self‐drying ILs protect the formed polycations from hydrolysis. The structures of the polycationic compounds can be tuned by using different Lewis acids.

**Figure 9:**
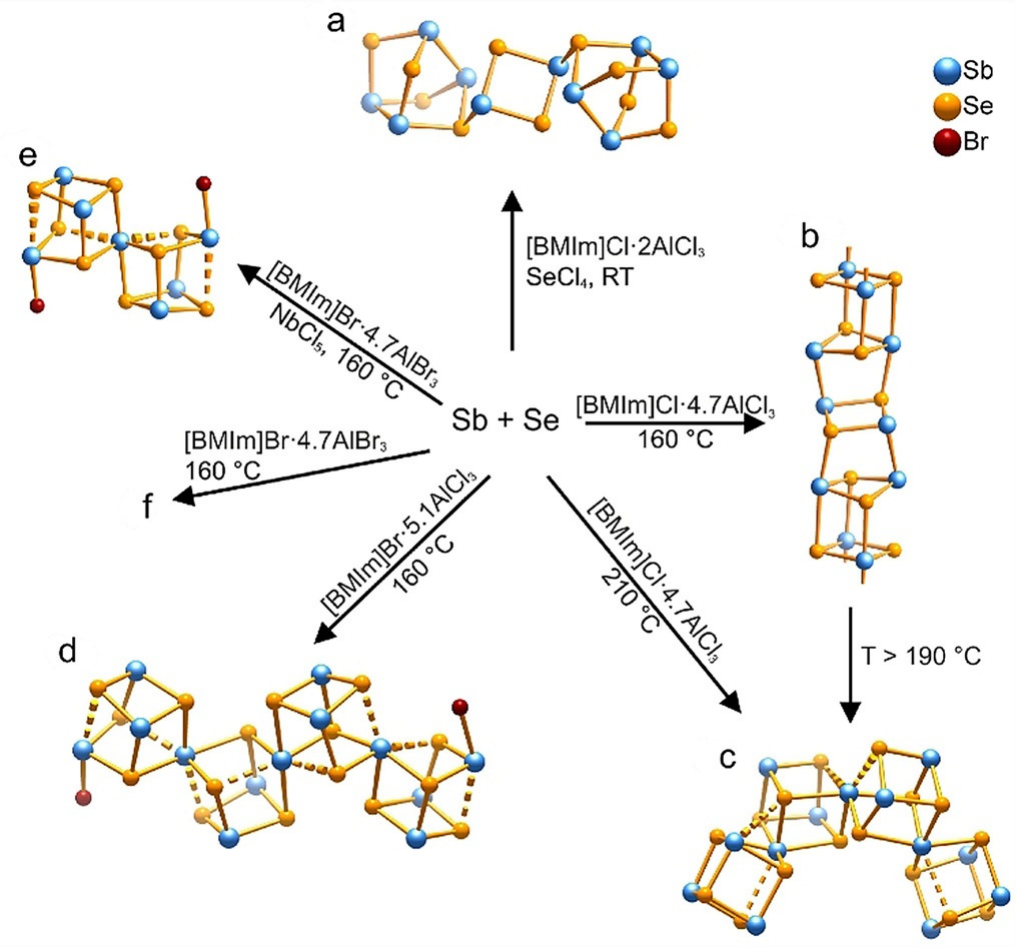
Structures of Sb‐Se heteropolycations obtained in Lewis‐acidic ILs. Copyright 2016, MDPI: Multidisciplinary Digital Publishing Institute.^[13]^ a) [Sb_10_Se_10_]^2+^ in [Sb_10_Se_10_][AlCl_4_]_2_;^[166]^ b) 1∞ [Sb_2_Se_2_]^+^ in [Sb_2_Se_2_][AlCl_4_];^[167]^ c) [Sb_13_Se_16_]^7+^ in [Sb_13_Se_16_][AlCl_4_]_6_Al_2_Cl_7_;[^167^, ^168^] d) [Sb_13_Se_16_Br_2_]^5+^ in [Sb_13_Se_16_Br_2_][Al*X*_4_]_5_;^[169]^ e) [Sb_7_Se_8_Br_2_]^3+^ in [Sb_7_Se_8_Br_2_][Al*X*_4_]_3_;^[169]^ and f) [Sb_7_Se_8_Br_2_]^3+^ and [Sb_13_Se_16_Br_2_]^5+^ coexisting in [Sb_7_Se_8_Br_2_][Sb_13_Se_16_Br_2_][AlBr_4_]_8_.^[169]^.

For example, Ruck and co‐workers reported that several Sb‐Se heteropolycations can be accessed in the Lewis‐acidic room‐temperature ILs [BMIm]X⋅*n* AlX_3_ (X=Br, Cl; *n*=1.2–5.2) (Figure 9).[^166^, ^167^, ^168^, ^169^] If Lewis‐acidic ILs [BMIm]Cl⋅*n* AlCl_3_ are used in the synthesis, some binary Sb‐Se polycations are obtained. Reactions of Sb with Se in [BMIm]Cl⋅4.7 AlCl_3_ yield, depending on the reaction temperature, the two cluster compounds [Sb_2_Se_2_][AlCl_4_] and [Sb_13_Se_16_][AlCl_4_]_6_Al_2_Cl_7_.[^167^, ^168^] When SeCl_4_ is added to Se and Sb in [BMIm]Cl⋅*n* AlCl_3_, [Sb_10_Se_10_][AlCl_4_]_2_ crystallizes at room temperature.^[166]^ If [BMIm]Br⋅*n* AlX_3_, however, is used as the reaction medium, ternary Sb‐Se‐Br polycations with the general formula [Sb_4+3*n*_Se_4+4*n*_Br_2_]^(2+*n*)+^ are produced.^[169]^ These cationic clusters are spiro‐heterocubanes with two terminal bromide ions, and their structures can be tuned by varying the bromine‐to‐chlorine ratio in the ILs. The use of the chlorine‐rich IL [BMIm]Br⋅5.1 AlCl_3_ at 160 °C results in the precipitation of [Sb_13_Se_16_Br_2_][AlX_4_]_5_. However, [Sb_7_Se_8_Br_2_][Sb_13_Se_16_Br_2_][AlBr_4_]_8_ crystallizes if the purely bromine IL [BMIm]Br⋅4.7 AlBr_3_ is used for the reaction. Finally, [Sb_7_Se_8_Br_2_][AlX_4_]_3_ is produced in [BMIm]Br⋅4.7 AlBr_3_ in the presence of a small amount of NbCl_5_. NbCl_5_ is added to modify the Lewis acidity.

In another synthesis, the two compounds (CuBi_8_)[AlCl_4_]_2_[Al_2_Cl_7_] and (CuBi_8_)[AlCl_4_]_3_ were accessed by reacting stoichiometric amounts of Bi, BiCl_3_, with CuCl in the IL [BMIm]Cl⋅4 AlCl_3_ at 180 °C; (CuBi_8_)^3+^ is the first intermetallic bismuth polycation with a 3d metal atom.^[170]^ The Lewis‐acidic IL takes part in the reaction, whereby [AlCl_4_]^−^ or/and the [Al_2_Cl_7_]^−^ ion becomes part of the final product to balance the (CuBi_8_)^3+^ cluster cation. Moreover, one of the chloride ions of the [AlCl_4_]^−^ group coordinates to the copper atom, thereby completing its 18‐electron count. Further investigations have shown that the (CuBi_8_)^3+^ cluster cannot be obtained either in molten AlCl_3_ or in ILs containing deficient/excess AlCl_3_, thus showing the indispensable role of the IL and a suitable Lewis acid content.

## 7 Reactions of Deep Eutectic Solvents

DESs exhibit some similar physical and chemical properties as ILs. Thus, they also lead to significant successes in inorganic synthesis, especially in large‐scale applications because of their inexpensive constituents and easy preparation. This DES‐based synthetic strategy opens up many new opportunities for the synthesis of zeolites and other inorganic nanomaterials. In many cases, however, one or more components can decompose in the DESs upon moderate heating. This section summarizes some examples of the preparation of inorganic materials by utilizing the unstable properties of DESs. An overview is given in Table 3.

**Table 3:** A summary of all the studies on inorganic materials prepared in DESs discussed in this review.

Materials composition	Deep eutectic solvents	Refs.
Al_2_(PO_4_)_3_⋅3 NH_4_	choline chloride/urea	[6]
[Zn(O_3_PCH_2_CO_2_)]⋅NH_4_	choline chloride/urea	[172]
Al(PO_4_)_2_⋅(CH_3_NH_3_)_2_(NH_4_), Al(HPO_4_)_2_F⋅(CH_3_NH_3_)_2_,	tetraethylammonium bromide (choline chloride)/1,3‐dimethyl urea	[173]
Al(PO_4_)_2_⋅(NH_3_(CH_3_)_2_NH_3_)(NH_4_), Al(PO_4_)_2_⋅(NH_3_(CH_3_)_2_NH_3_)(NH_4_)	choline chloride/ethylene urea	[173]
Al_3_(PO_4_)_4_⋅*n*((NH_3_(CH_2_)_3_NH_3_), Al(HPO_4_)(PO_4_)(NH_3_(CH_2_)_3_NH_3_), Al_3_(PO_4_)_4_⋅*n*((NH_3_(CH_2_)_3_NH_3_), Al_2_(OH)(PO_4_)_2_⋅2 H_2_O⋅NH_4_	choline chloride/*N*,*N*′‐trimethylene urea	[173]
[Al_3_CoClP_4_O_16_][C_5_H_13_NOH]_2_	choline chloride/succinic acid, choline chloride/glutaric acid, or choline chloride/citric acid	[177]
C_5_H_14_NO⋅ZnCl(HPO_3_)	choline chloride/urea	[180]
Ni(NH_3_)_6_Cl_2_, NiCl_2_, α‐Ni(OH)_2_, NiO	choline chloride/urea	[181]
α‐Co(OH)_2_, Co_3_O_4_	choline chloride/urea	[182]
Fe_2_O_3_	choline chloride/urea	[183]
MnCO_3_	choline chloride/urea	[171]
CoFe layered double hydroxide and CoFe oxide	choline chloride/urea	[184, 185]
Bi_2_S_3_, Sb_2_S_3_, CuS, ZnS, PbS, Ag_2_S, and CdS	choline chloride/thioacetamide	[186]
iron alkoxide of glycerol	choline chloride/glycerol	[187]

To date, the 1:2 mixture of choline chloride and urea (ChCl/urea, melting point 12 °C) is the most widely investigated DES in the literature. Investigations have shown that heating at 125–225 °C usually leads to decomposition of the urea in ChCl/urea, thereby resulting in the reactivity of ChCl/urea at high temperatures.^[171]^ Thus, ChCl/urea is usually used as a reactive reagent for the preparation of various inorganic compounds with unusual structures. In 2004, Morris and co‐workers first reported that a novel zeolite‐type framework (SIZ‐2, Al_2_(PO_4_)_3_⋅3 NH_4_) was produced in a ChCl/urea eutectic mixture (Figure 7 b).^[6]^ The ammonia stemming from the partial decomposition of the urea templates the structure and balances the charge of the framework that forms the interrupted structure of SIZ‐2. Similarly, a new zinc organophosphate was synthesized in a ChCl/urea mixture by Liao et al. and, again, the ammonia acts as a template.^[172]^ Later, Morris and co‐workers studied several eutectic mixtures based on quaternary ammonium halides (e.g. choline chloride and tetraethylammonium bromide) and urea derivatives (e.g. 1,3‐dimethylurea, ethylene urea, and *N*,*N*′‐trimethyleneurea) as the reaction media for the ionothermal synthesis of new zeolites.^[173]^ As expected, the breakdown of the various urea derivatives of the DESs at high temperatures gives rise to the corresponding organic species (e.g. methylammonium, ethylene diammonium, and propylene diammonium), which serve as templates and enable controlled delivery to the reaction mixture. Nine aluminophosphate materials including five unknown compounds have been prepared in this way.

Metal phosphate MPO_4_ (M=Ga, Zr, Co, Fe, and Mn) frameworks can also be successfully synthesized using various DESs.[^174^, ^175^, ^176^] The organic template is delivered to the reaction mixture by decomposition of one or more components of the DES mixture. Some new metal phosphate frameworks have been produced using the unique and flexible properties of DESs.

Clearly, in these ChCl/urea‐based reaction systems, it is the urea portion that provides the better template. In fact, choline itself can be a very attractive template. To avoid the competition between these two ammonium cations as templates, Morris and co‐workers synthesized a series of DESs based on choline chloride/carboxylic acid for the ionothermal synthesis of cobalt aluminophosphate (CoAlPO) materials,^[177]^ including an unusual layered zeolite material ([Al_3_CoClP_4_O_16_][C_5_H_13_NOH]_2_, SIZ‐13). The choline cations fill the interlayer space without significant chemical modification. However, the chloride ions from choline chloride are incorporated into the structure of SIZ‐13 through the formation of covalent Co−Cl bonds. Such metal–chlorine bonds have not been found in the hydrothermal synthesis because of their sensitivity to hydrolysis, but the water in DESs tends to be less reactive because of strong interactions with anions of the DESs.[^178^, ^179^] Similarly, Harrison reported that the compound C_5_H_14_NO⋅ZnCl(HPO_3_), which contains covalent Zn−Cl bonds, was obtained by reacting choline chloride with Zn^2+^ and hydrogen phosphite precursors in ChCl/urea.^[180]^

**Figure 10:**
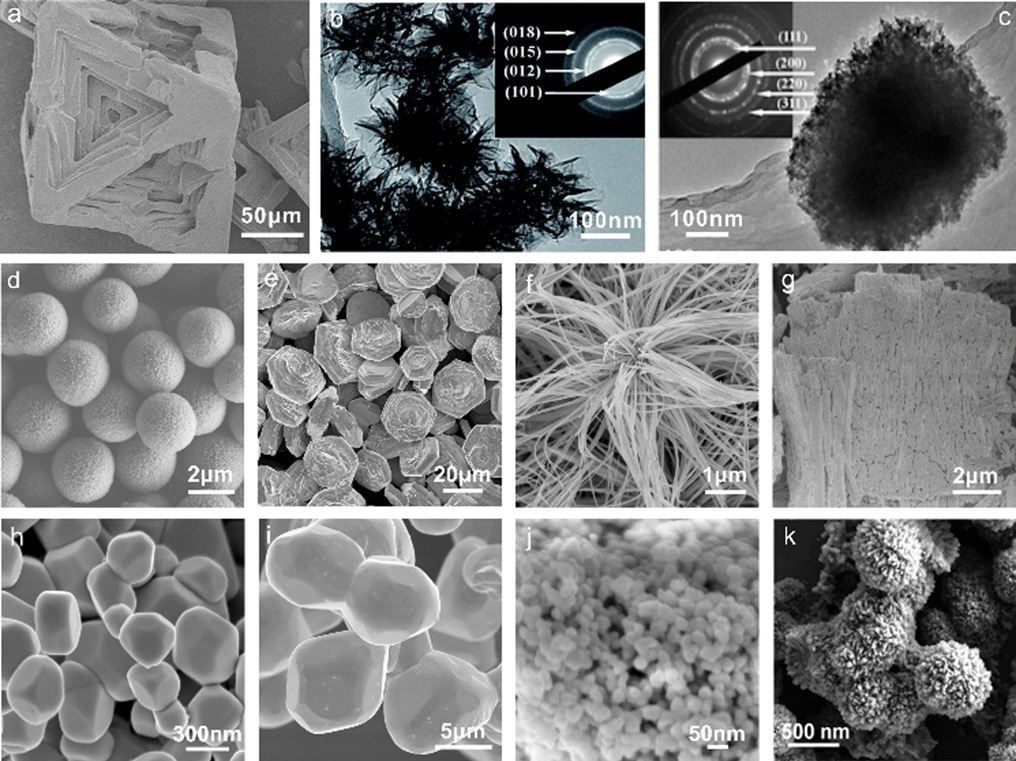
a) SEM image of the Ni[NH_3_]_6_Cl_2_ octahedron with exfoliated facets. b,c) TEM image and corresponding SEAD patterns of α‐Ni(OH)_2_ and NiO. Reproduced with permission.[^39^, ^181^] Copyright 2013 and 2017, Royal Society of Chemistry. d–j) SEM images of ZnS, CuS, Bi_2_S_3_, Sb_2_S_3_, PbS, Ag_2_S, and CdS, respectively, which are obtained from ChCl/TAA‐based DESs. Reproduced with permission.^[186]^ Copyright 2017, Wiley‐VCH. k) SEM image of the as‐synthesized iron glycerate hybrid in ChCl/glycerol based DES. Reproduced with permission.^[187]^ Copyright 2019, American Chemical Society.

DESs are also applied as reactive reagents for the preparation of various functional materials such as metal hydroxides, metal oxides, metal chalcogenides, and organic–inorganic hybrids.^[39]^ Gu and co‐workers synthesized various nanostructured transition‐metal complexes and layered transition‐metal hydroxides, as well as their derivatives in ChCl/urea through an ionothermal strategy at a relatively high temperature (120–210 °C). Octahedral [Ni(NH_3_)_6_]Cl_2_ crystals with an open structure can be obtained when the Ni^2+^:ChCl/urea solution is heated in a sealed vessel (Figure 10 a).^[181]^ Nanosheet‐like NiCl_2_ is produced by annealing the [Ni(NH_3_)_6_]Cl_2_ precursor. When the Ni^2+^:ChCl/urea solution is thermally treated under an open system, however, the ammonia released from the urea is removed from the reaction solution, which leads to the formation of the flower‐like α‐Ni(OH)_2_ when a small amount of water is added to the solution under heating (Figure 10 b). NiO with the same flower‐like morphology is synthesized through annealing the as‐obtained α‐Ni(OH)_2_ (Figure 10 c). Similarly, α‐Co(OH)_2_ and Co_3_O_4_ can also be accessed by this water injection method.^[182]^ In the case of Fe, however, it is the Fe‐oxide phase Fe_2_O_3_ that is directly obtained from a Fe^3+^:ChCl/urea solution.^[183]^ If a Mn^2+^:ChCl/urea solution is heated in a closed system, a calcite‐type MnCO_3_ phase is produced.^[171]^ Moreover, a “two‐stage water injection” strategy is applied to synthesize the cobalt iron layered double hydroxide (CoFe LDH) with different interlayer spacings.^[184]^ Investigations have shown that the volume of injected water at each stage plays an important role in determining the structure of the CoFe LDH. When a small amount of water is injected in the first stage, the ChCl‐DES maintains its superstructure. In this case, CoFe LDH with an expanded interlayer spacing of 11.3 Å in its (003) plane is obtained. Further calcination of the CoFe LDHs results in porous CoFe oxide nanosheets with a large specific surface area of 79.5 m^2^ g^−1^.^[185]^

In addition to the well‐studied ChCl/urea‐based DES, other types of DESs may also be used as reactive reagents for the formation of inorganic materials. A family of binary metal sulfides, such as Bi_2_S_3_, Sb_2_S_3_, CuS, ZnS, PbS, Ag_2_S, and CdS, have been successfully synthesized by Ruck and co‐workers by using a DES based on ChCl/thioacetamide (TAA; Figure 10 d—j).^[186]^ The proposed reaction mechanism consists of: 1) a metal salt is dissolved or dispersed in the ChCl/TAA‐based DES and the corresponding metal‐DES is formed; 2) the final sulfide is formed by thermal decomposition of the metal–DES complex. This ChCl/TAA‐based DES serves as both the solvent and sulfur source, providing an ideal “all‐in‐one” reaction medium for the efficient synthesis of sulfide nanoparticles.

Li and co‐workers found that a Fe‐based organic–inorganic hybrid—a hierarchical 3D iron alkoxide of glycerol—was synthesized using Fe(NO_3_)_3_⋅9 H_2_O as a starting material in a ChCl/glycerol (1:2) DES (Figure 10 k).^[187]^ The ChCl/glycerol DES serves not only as a benign reaction medium but also as a reactant. Fe^3+^ is chelated by glycerol and is integrated into the DES matrix through coordinative bonding and hydrogen bonding. Thus, the reactants are effectively brought together by the DES through a prestructuring effect, which results in the formation of the 3D hierarchical seedlike iron alkoxide of glycerol nanospheres. The as‐synthesized Fe‐based organic‐inorganic hybrid exhibits an enhanced oxygen evolution reaction performance, with a low overpotential of 280 mV at a current density of 10 mA cm^−2^.

## 8 Other Types of Reactions of Ionic Liquids

In some IL‐based reactions, the IL itself may undergo complete decomposition. As mentioned in Section 5, phosphonium ILs are partly decomposed into trialkylphosphanes, and these intermediates then react with Se/Te to form the corresponding trialkylphosphane selenides/tellurides. Interestingly, phosphonium ILs can be further used as a phosphorus source for the synthesis of metal phosphides through complete decomposition of the quaternary phosphonium cations at higher temperatures (>350 °C). Li and co‐workers reported that nanostructured Ni_2_P and Ni_12_P_5_ nanoparticles were fabricated using [P_4444_]Cl as both the phosphorus source and reaction medium upon microwave heating at 350 °C for 1–2 minutes.^[188]^ The as‐synthesized Ni_2_P nanocrystals show an enhanced electrocatalytic hydrogen evolution performance in an acidic medium. Moreover, phosphonium ILs can be designed to be metal‐containing ILs and can thus be utilized as both the metal and phosphorus source for the preparation of metal phosphides. For example, [P_66614_]_2_[CoCl_4_] was used to synthesize Co_2_P by a one‐step phosphidation at 400 °C without adding other reagents.[^189^, ^190^] The obtained Co_2_P/carbon nanotube (CNT) composite shows high activity for the hydrogen evolution reaction. This strategy based on phosphonium ILs provides a remarkable advantage for the efficient synthesis of metal phosphides. Ruck and co‐workers reported the synthesis of copper‐deficient Cu_3−*x*_P (0.1<*x*<0.7) from elemental precursors in halide ILs (e.g. [P_66614_]Cl).[^42^, ^191^] Investigations have shown that the halide anions drastically promote the reactivity of red or white phosphorus and kinetically suppress the formation of Cu_2_P by‐products. Based on mechanistic studies, it was found that chloride ions act as strong nucleophiles that attack the phosphorus network, thereby resulting in degradation of the phosphorus. At a high concentration of chloride ions, the P−P bonds are sufficiently activated, leading to a drastic increase in the formation of the Cu_3−*x*_P phase.

Recently, there has been a lot of interest in using ILs (including DESs) as versatile carbon precursors, rather than conventional polymers, for the preparation of carbon materials because of the unique properties of ILs, such as negligible vapor pressure, carbon‐rich nature, and structural diversity. In the carbonization processes, ILs are completely decomposed, and the corresponding ILs are converted into carbon residues. The yield, properties, and structure of the obtained carbons depend on the structure of the IL precursors. The relevant studies have been comprehensively reviewed elsewhere,[^5^, ^39^, ^192^, ^193^] and will not be the focus of this Review.

## 9 Summary and Outlook

Great developments have been made in inorganic syntheses using, or in the presence of, ILs, which has led to the formation of diverse compounds with interesting properties. However, the chemical reactivity of the ILs and DESs in the reactions is usually neglected or not explicitly discussed. This Review gives an overview of the chemical reactions of ILs or DESs in inorganic synthesis.

Metal‐containing ILs represent a promising class of metal sources. The IL properties can be tailored by varying the ligand structures and the incorporated metal ions, which enables the formation of inorganic nanoparticles with diverse sizes and morphologies. Basic ILs are usually employed to replace the traditional bases, such as NaOH, KOH, or Na_2_CO_3_, to provide the required basic environment for the production of metal (hydr)oxide particles. Moreover, the organic cations of the basic ILs can act as a template to control the crystal nucleation and growth. The hydrolysis of [BF_4_]^−^ or [PF_6_]^−^ anions gives a fluorine source for the synthesis of metal fluorides, thereby avoiding the use of toxic and harmful HF, NaF, or NH_4_F. The C2‐position of the 1,3‐dialkylimidazolium cation shows reactivity towards chalcogens (e.g. S, Se, and Te) for the formation of the corresponding imidazole‐2‐chalcogenones, especially in the presence of a base. In addition, phosphonium ILs can react with chalcogens at high temperatures to generate the corresponding trialkylphosphane chalcogenides. These formed imidazole‐2‐chalcogenones or trialkylphosphane chalcogenides can be further applied as chalcogen sources for the preparation of metal chalcogenides. When some porous materials such as zeolites, MOFs, and polycationic/polyanionic compounds are synthesized by an ionothermal approach, either the cation or anion of the IL can be incorporated in the channels of the products to balance the charged frameworks and can also serve as structural templates. In the case of DESs, one or more of their components may decompose to form in situ templates for the formation of metal phosphate frameworks or act as reactive species for the synthesis of functional nanoparticles (e.g. metal hydroxides, metal oxides, metal chalcogenides, and organic–inorganic hybrids).

The chemical reactivity of ILs and DESs in inorganic synthesis should not be underestimated. Furthermore, the reactive properties of the ILs and DESs in the reactions can be used as a synthetic tool to prepare inorganic materials that are difficult or even impossible to obtain by traditional synthetic routes. In this regard, it is important to have an in‐depth understanding of the interaction of ILs or DESs with reactants and solutes, and thus fully understand the reaction mechanism for the directed use of ILs and DESs for the preparation of inorganic materials.

## Conflict of interest

The authors declare no conflict of interest.

## Biographical Information

*Tao Zhang obtained his PhD in inorganic chemistry from the Technische Universität Dresden in 2018 under the supervision of Prof. Michael Ruck. He then joined to the Institute of Process Engineering, Chinese Academy of Sciences in 2019 as an Associate Professor. His research interests are concerned with the synthesis of functional materials in ionic liquids*.

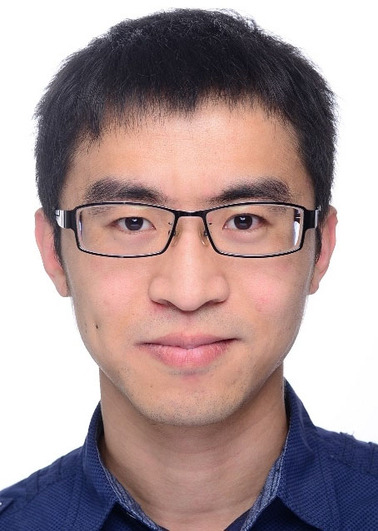

## Biographical Information

*Thomas Doert received his PhD from the University of Düsseldorf in 1994 with Prof. Peter Böttcher. He then moved to the Technische Universität Dresden, where he is now Adjunct Professor. He was visiting scientist at Stockholm University as well as Guest Professor at Strasbourg University and Shanghai University of Engineering Science. His research interests cover crystallographic aspects of solid‐state chemistry, solid‐state chemistry of chalcogenides, layered materials, and frustrated magnets*.

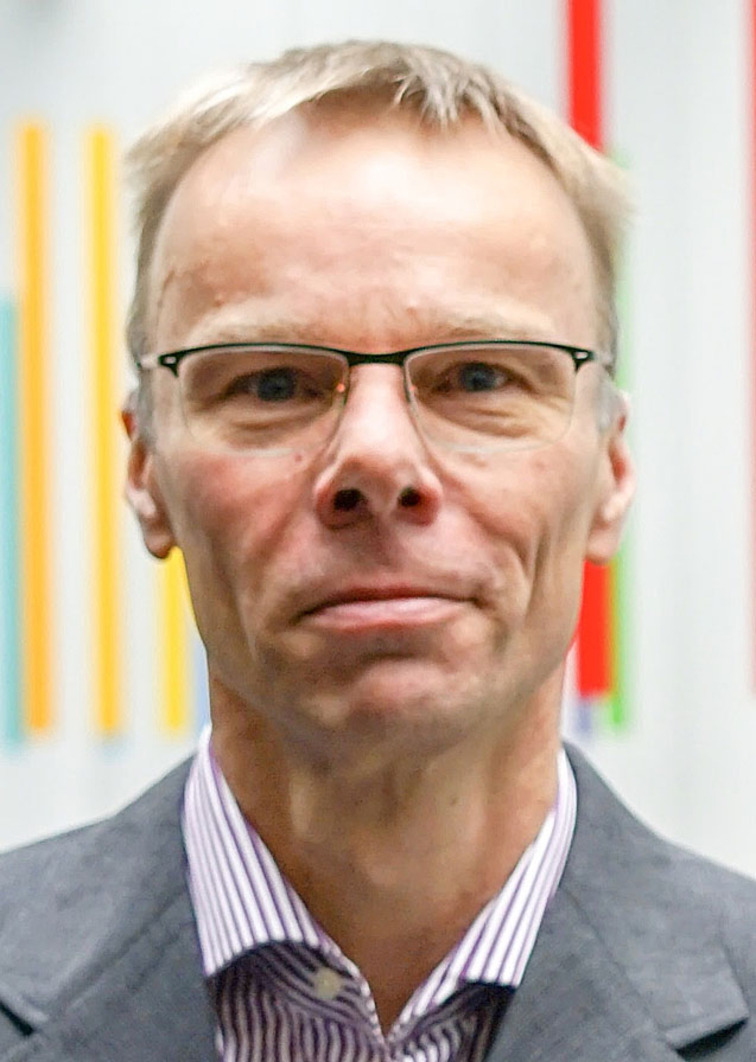

## Biographical Information

*Hui Wang is a professor at the Institute of Process Engineering of the Chinese Academy of Sciences. She received her PhD from the University of the Chinese Academy of Sciences in 2010 with Prof. Zengxi Li. In the same year, she joined Professor Robin D. Rogers’ Group at the University of Alabama as a postdoctoral research fellow. In 2015, she moved to the Institute of Process Engineering, Beijing. Her research interests focus on the properties of ionic liquids and the applications of ionic liquids in green chemical processes*.

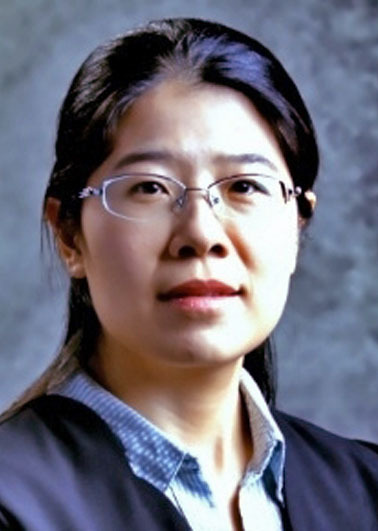

## Biographical Information

*Suojiang Zhang received his PhD from Zhejiang University in 1994 with Prof. Shijun Han. He has been a professor at the Institute of Process Engineering of the Chinese Academy of Sciences since 2001, and was elected as a member of the Chinese Academy of Sciences in 2015. He is currently Director General of the Institute of Process Engineering and Fellow of the Royal Society of Chemistry. His research interests focus on the designed synthesis of ionic liquids and their applications in green chemistry and green process engineering*.

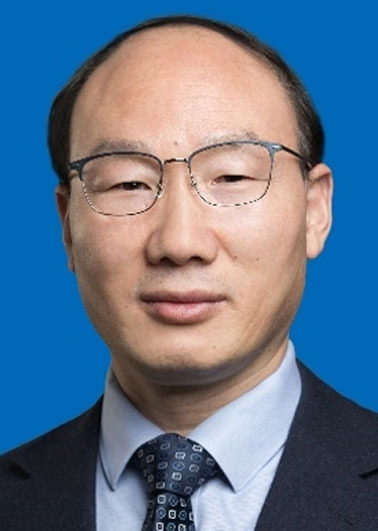

## Biographical Information

*Michael Ruck received his PhD from the University of Stuttgart in 1991 with Prof. Arndt Simon. After his habilitation at the University of Karlsruhe in 1997, he was a Heisenberg fellow of the German Science Foundation. Since 2000 he has been Full Professor of Inorganic Chemistry at the Technische Universität Dresden, where he has served as Dean of the Faculty of Science and Vice Rector. He has repeatedly been appointed a Fellow of the Max Planck Society and awarded the Steinhofer Research Award and the Will Kleber Commemorative Coin. His research interests include solid‐state chemistry and sustainable material syntheses*.